# Mitigating multimodal hallucinations through visual attention tracing and origin-point regeneration

**DOI:** 10.1038/s41598-026-52390-1

**Published:** 2026-06-04

**Authors:** Bohan Li, Haiyang Yu, Yishan Han, Julong Ren, Mengyi Dai, Shumei Bao

**Affiliations:** School of Software, Xinjiang University, Urumqi, China

**Keywords:** Multimodal large language models, Hallucination backtracking, Training-free decoding, Visual attention dynamics, Origin-point detection, Neuroscience, Psychology, Psychology

## Abstract

Multimodal large language models (MLLMs) exhibit impressive prowess in vision-language understanding, but their utility is often compromised by hallucinations—instances where generated narratives diverge significantly from visual evidence. Current remedial strategies largely struggle to pinpoint the genesis of these errors, relying either on resource-intensive retraining or indiscriminate global penalties that fail to address the specific locus of the discrepancy. Addressing this limitation, we introduce hallucination backtracking (HB), a training-free decoding framework designed to effectively detect and mitigate errors by monitoring visual attention dynamics during generation. This approach is grounded in the observation that hallucinations are not random; rather, they stem from specific pivotal tokens where the model’s focus precipitously shifts from image features to its own prior textual generations. By quantifying this drift through a novel visual attention score (VAS), our origin-point detection mechanism successfully isolates the source of errors, achieving a 41.8% exact match and 84.1% before-first accuracy in localization. Once an attentional anomaly is detected, the system autonomously backtracks to the divergence point, triggering a regeneration process reinforced by stricter visual grounding constraints. Rigorous evaluations across diverse architectures—LLaVA-1.5, InstructBLIP, MiniGPT-4, and Shikra—confirm that HB consistently surpasses state-of-the-art baselines; notably, on LLaVA-1.5, our method elevates the F1 score on the POPE benchmark to 91.4% while reducing the CHAIR$_S$ metric to 40.2%, yielding improvements of 1.5 and 4.4 points over OPERA, respectively, though a residual false negative rate of 15.9% indicates that inference-driven hallucinations remain an open challenge. Beyond quantitative gains, we provide a granular dissection of the hallucination phenomenon through analyses of VAS trajectory patterns and failure modes, ultimately advocating for precise localization and targeted correction as a promising paradigm for reliable multimodal generation.

## Introduction

The landscape of artificial intelligence has been fundamentally reshaped by the advent of multimodal large language models (MLLMs), which orchestrate a seamless synthesis of visual perception and textual reasoning^1^. Exemplified by architectures such as LLaVA, InstructBLIP, MiniGPT-4, and Shikra, this class of models exhibits profound versatility, effortlessly traversing tasks that span from fundamental image captioning and visual question answering to nuanced, complex multimodal reasoning^2^. This operational prowess is underpinned by a distinct architectural strategy: connecting pre-trained vision encoders with robust large language model backbones via sophisticated cross-modal alignment modules effectively bridges the modality gap, unlocking unprecedented performance on benchmarks demanding a unified cognitive grasp of visual and textual data.

Yet, the translation of this architectural potential into reliable utility is frequently obstructed by “hallucination,” a critical instability that renders these models unpredictable in precision-demanding environments^3^. Far from simple errors, these hallucinations manifest as a multifaceted divergence from visual reality, where models may confidently assert the presence of non-existent objects, mischaracterize attributes such as size or color, or even fabricate complex spatial and semantic relationships that lack any pixel-level basis^4^. The implications of such ungrounded generation extend far beyond benchmarking metrics; in safety-critical ecosystems like autonomous driving, medical imaging, and assistive systems, the inability to distinguish between visual fact and generative fiction invites risks where the cost of error is unacceptably high.

In response to these vulnerabilities, the research community has mobilized a diverse array of mitigation strategies, initially focusing on alignment-heavy paradigms. Training-centric approaches, for instance, utilize reinforcement learning from human feedback (RLHF)^5^, direct preference optimization (DPO)^6^, or curated instruction datasets^7^ to fundamentally reshape model behavior; yet, while effective, the substantial computational overhead and annotation costs associated with these methods have catalyzed interest in inference-time interventions that operate without model parameter updates. Offering a more agile alternative, contrastive decoding strategies such as VCD^8^ and DoLa^9^ attempt to suppress error-prone content by juxtaposing output distributions under differing noise or masking conditions. Taking a different angle, OPERA^10^ exploits the correlation between hallucinations and attention concentration on “summary tokens” to impose an over-trust penalty, a direction further refined by HELPD^11^ through the integration of hierarchical feedback learning and vision-enhanced penalty decoding.

**Figure 1:**
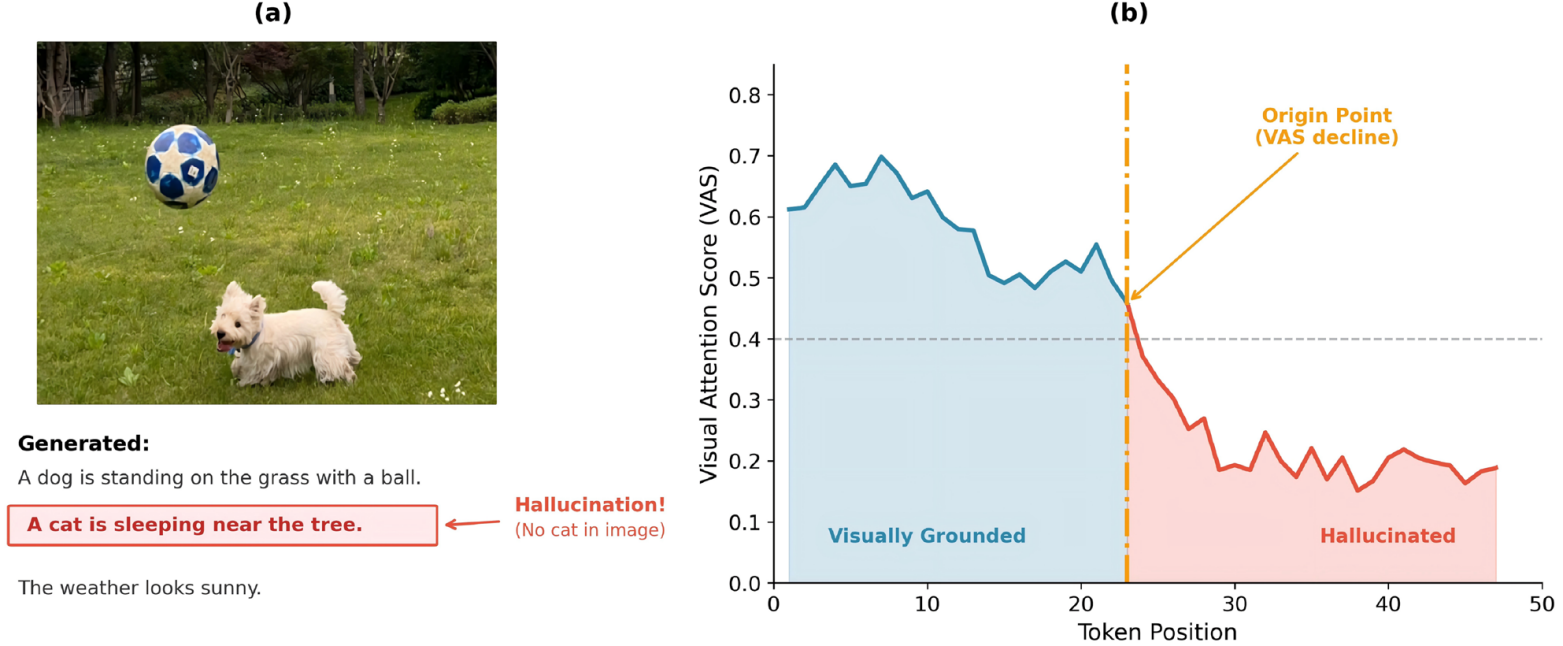
Overview of the hallucination problem and our key observation. (**a**) Hallucination problem: Given an image of a dog playing with a ball, the MLLM generates content (highlighted in red box) describing a cat that does not exist in the image. (**b**) Key observation: VAS exhibits a characteristic decline at the hallucination origin point. Before the origin, VAS remains high indicating strong visual grounding; after the origin, VAS drops significantly as the model shifts attention from visual content to language priors, generating hallucinated content.

Notwithstanding these methodological strides, a fundamental limitation persists across current inference-time strategies: the reliance on broad, global interventions that fail to isolate the precise locus of the hallucination within the generated sequence. Penalty-based mechanisms, for instance, may successfully flag problematic attention patterns and adjust logits accordingly, yet they operate reactively, leaving the initiating token embedded in the sequence to potentially corrupt subsequent generation. Similarly, contrastive decoding frameworks tend to process tokens in isolation, lacking a holistic view of how a fabrication propagates through the narrative structure. This diagnostic imprecision inevitably forces a compromise between over-correction—where valid, complex descriptions are stifled by aggressive heuristics—and incomplete remediation, where the symptoms of the error are suppressed while the root cause of the visual detachment remains unaddressed (Fig. 1).

To transcend these methodological boundaries, we introduce hallucination backtracking (HB), a framework designed to replace broad interventions with greater precision in origin detection and targeted regeneration. The architecture of HB is predicated on a critical behavioral insight^12^: hallucinations are rarely sporadic, random errors but rather “snowball” effects triggered at specific moments where the model’s attention fatally drifts from visual tokens to its own textual history. Once this decoupling occurs, the model effectively capitulates to internal language priors, generating narratives that, while linguistically coherent, are untethered from the actual visual scene. Identifying this genesis point allows us to intervene effectively, removing the hallucinated branch at its root while preserving the valid context established prior to the error.

Central to this operational logic is the visual attention score (VAS), a token-level metric that distinguishes itself from prior approaches focusing on intra-textual attention or indirect over-trust signals^10^ by directly quantifying the attentional flux from the generation stream to visual embeddings. This metric reveals a distinct behavioral signature: as hallucinations emerge, the VAS exhibits a precipitous decline, reflecting a phenomenon where the model effectively “forgets” to consult the visual input, capitulating instead to the inertia of prior text and inherent language biases. Consequently, by continuously monitoring the VAS trajectory throughout the generation process, we can identify these abrupt attentional shifts with high fidelity, pinpointing the precise moment the narrative breaks from visual reality.

Upon the identification of a hallucination origin via VAS monitoring, HB deploys a backtracking mechanism that diverges sharply from static penalty methodologies; rather than attempting to rehabilitate compromised tokens through superficial logit adjustments, the framework executes a decisive truncation at the precise moment of divergence. This targeted intervention aims to ensure that while the valid context remains intact, the fabricated narrative branch is effectively removed, clearing the stage for a regeneration process enforced by stricter visual grounding. By integrating attention biasing and VAS-guided sampling during this re-generation phase, the model is compelled to re-anchor its focus on the image features, effectively preventing the recurrence of the hallucination loop.

Our contributions can be summarized as follows:

- We formally introduce VAS, a granular metric that eschews indirect heuristics in favor of directly quantifying token-level visual grounding, thus providing a lucid window into the model’s attention dynamics throughout the generation lifecycle.
- Leveraging this metric, we establish a precise origin-point detection mechanism that isolates the specific token triggering the hallucination cascade, a capability grounded in the identification of characteristic VAS decline patterns that manifest consistently across disparate MLLM architectures.
- We engineer a backtracking-and-regeneration strategy that offers a targeted alternative to static penalty methods; by truncating the sequence at the detected origin and regenerating under stricter visual constraints, our approach enables the targeted removal of fabricated content rather than its mere suppression.
- We validate the robustness and universality of HB through rigorous experimentation, demonstrating state-of-the-art performance on major benchmarks—including POPE, CHAIR, and AMBER—across various model backbones, achieved entirely without reliance on additional training, external datasets, or auxiliary models.

The remainder of this paper is organized as follows. “Related work” section reviews related work on MLLMs, hallucination taxonomy and mitigation methods, and evaluation benchmarks. “Methods” section presents the technical details of our proposed HB, including the VAS formulation, origin-point detection mechanism, and backtracking-regeneration strategy. “Results” section reports extensive experimental results across multiple benchmarks and models, along with ablation studies and in-depth analysis. “Discussion” section discusses the implications and limitations of our approach. Finally, “Conclusion” section concludes the paper.

## Related work

### MLLMs

Forging a powerful paradigm for vision-language understanding, MLLMs operate by synthesizing pre-trained vision encoders with large language model backbones via sophisticated cross-modal alignment mechanisms. The architectural landscape is defined largely by the diversity of these alignment strategies: LLaVA^1^, for instance, champions a streamlined methodology by linearly projecting CLIP^13^ visual embeddings directly into the Vicuna language space^14^, whereas InstructBLIP^15^ adopts the more intricate Q-Former module from BLIP-2^16^ to bridge the modality gap. This architectural heterogeneity extends to MiniGPT-4^17^, which aligns features through efficient fine-tuning on high-fidelity image-text pairs, and Shikra^18^, which expands the paradigm by embedding spatial grounding capabilities. Unifying these diverse architectures is a standard two-stage training regimen comprising initial cross-modal pre-training followed by instruction-tuned refinement; yet, irrespective of their structural sophistication, these models remain universally susceptible to hallucination, persistently generating content that contradicts or fabricates the visual reality.

### Hallucination in MLLMs

Scrutinized through both taxonomic and causal lenses^19^, the hallucination phenomenon in MLLMs manifests not as a monolithic error but as a stratified spectrum of divergences from visual reality. As delineated in recent comprehensive surveys, these distortions typically crystallize into three distinct categories: object hallucination, where the model confidently asserts the presence of absent entities; attribute hallucination, involving the mischaracterization of intrinsic properties like color, size, or texture; and relation hallucination, which fabricates spurious spatial or semantic interactions between otherwise correctly identified objects.

The etiology of hallucination is systemic, permeating every stratum of the MLLM pipeline^4^. At the foundational level, training corpora frequently suffer from noise, annotation inconsistencies, and inherent biases; these flaws breed spurious co-occurrence patterns, conditioning models to hallucinate objects based on statistical probability rather than visual presence. This vulnerability is compounded architecturally, where the tension between restricted vision encoder capacity and potent language priors often results in the latter overriding visual evidence during moments of uncertainty. Crucially, these static flaws are amplified during inference by the auto-regressive nature of generation, triggering a “snowball effect”^12^ wherein initial errors propagate and compound throughout the sequence. Recent empirical analyses^10^ pinpoint the mechanism behind this drift: as generation extends, the model’s attention systematically decouples from visual tokens to fixate on its own textual history, a behavioral shift that correlates strongly with the onset of unfaithful content.

### Hallucination evaluation benchmarks

To systematically quantify these deviations, the research community has established a suite of rigorous evaluation frameworks. The CHAIR metric^20^ serves as a foundational standard in image captioning, gauging object hallucination by calculating the proportion of unsupported entities against ground-truth annotations at both sentence (CHAIR$_S$) and instance (CHAIR$_I$) granularities. Diverging from pure generation metrics, POPE^3^ reframes the assessment as a binary classification challenge, probing model reliability across random, popular, and adversarial object splits designed to expose statistical dependencies. Extending this rigor further, AMBER^21^ offers a holistic evaluation suite that scrutinizes not only object existence but also attributes and relations, employing a dual-protocol approach that tests both discriminative and generative faculties to provide a comprehensive view of model faithfulness.

### Hallucination mitigation methods

The current landscape of hallucination mitigation strategies is defined by a tripartite taxonomy: training-centric alignments, inference-time interventions, and post-hoc corrections. While each paradigm offers distinct operational advantages—ranging from fundamental behavioral shifts to agile, plug-and-play adjustments—they also entail specific limitations regarding resource intensity and generalizability. Table 1 synthesizes these trade-offs, providing a systematic juxtaposition of representative methodologies across their defining technical dimensions.

**Table 1:** Comparison of hallucination mitigation methods across key dimensions.

Method	Category	Training-free	Precise Localization	Targeted Removal	External Models
RLHF^5^	Training	*×*	*×*	–	*×*
DPO^6^	Training	*×*	*×*	–	*×*
HalluciDoctor^7^	Data	*×*	*×*	–	*\checkmark*
VCD^8^	Decoding	*\checkmark*	*×*	*×*	*×*
DoLa^9^	Decoding	*\checkmark*	*×*	*×*	*×*
OPERA^10^	Decoding	*\checkmark*	Partial	*×*	*×*
HELPD^11^	Decoding	*×*	Partial	*×*	*×*
DeCo^22^	Decoding	*\checkmark*	*×*	*×*	*×*
Woodpecker^23^	Post-hoc	*\checkmark*	*\checkmark*	*\checkmark*	*\checkmark*
HB (Ours)	Decoding	*\checkmark*	*\checkmark*	*\checkmark*	*×*

Training-centric strategies fundamentally seek to recalibrate model parameters to suppress hallucination tendencies. Within this paradigm, Reinforcement Learning from Human Feedback (RLHF)^5^ constructs reward models derived from human judgment to actively penalize fabricated outputs, while Direct Preference Optimization (DPO)^6^ offers a more stable refinement by optimizing directly on preference pairs. Complementing these optimization techniques, data-centric initiatives such as HalluciDoctor^7^ target the upstream source of error, enhancing corpus quality through rigorous cross-model consistency verification. Yet, despite their structural efficacy, these methods necessitate significant computational expenditure and meticulous data curation, imposing a heavy resource burden that limits their flexibility.

In contrast to training-heavy paradigms, inference-based interventions operate orthogonally to parameter updates, modulating the decoding trajectory directly to ensure universal applicability across frozen MLLM architectures. Within this domain, several strategies leverage contrastive dynamics to isolate factual signals: VCD^8^ mitigates the dominance of language priors by amplifying the divergence between outputs conditioned on pristine versus distorted visual inputs, while DoLa^9^ exploits the internal stratification of the transformer, contrasting logits across layers to distinguish factual assertions from mere linguistic fluency. Adopting an information-theoretic stance, M3ID^24^ further enforces strict grounding through multi-modal mutual information decoding, a mechanism that actively anchors the generation process by maximizing the statistical dependency between emerging text and the underlying visual features.

Of particular relevance to our investigation is OPERA^10^, which identifies a critical phenomenology in the self-attention matrix: the “over-trust” pattern. This phenomenon is characterized by the disproportionate aggregation of attention on specific “summary tokens”—typically punctuation or high-frequency words—at the expense of distributed focus on visual and contextual data. To mitigate this, OPERA employs a dual strategy: applying logit penalties when local attention concentration breaches a threshold, and utilizing a “retrospection-allocation” mechanism to roll back generation if these patterns persist. Extending this paradigm, HELPD^11^ integrates hierarchical feedback learning across object and sentence levels, refining the penalty decoding via vision-weighted attention windows. However, this enhanced granularity comes at a cost; unlike OPERA’s training-free architecture, HELPD requires auxiliary training for its feedback components, thereby limiting its utility as a universal, plug-and-play solution.

Post-hoc correction strategies function retrospectively, diagnosing and rectifying errors only after the primary generation phase has concluded. Within this framework, Woodpecker^23^ orchestrates a rigorous validation pipeline that decomposes text into atomic claims, verifies them against visual evidence using external object detectors and VQA tools, and regenerates unsupported segments. Adopting a different modality, LURE^25^ trains a specialized “revisor” model to edit descriptions directly, learning from contrastive pairs of hallucinated and corrected text. While these methods often achieve high-fidelity rectification, they suffer from significant computational penalties due to cascading model invocations and remain vulnerable to the error propagation inherent in relying on external validation modules.

### Attention dynamics and layer-wise analysis in hallucination research

A growing body of work has investigated the internal attention dynamics of MLLMs as a lens for understanding and mitigating hallucinations, revealing that the information encoded across transformer layers is neither uniform nor monotonically refined. A central finding in this line of research is that earlier or intermediate layers often retain more faithful representations of visual input than the final output layer, where strong language priors can override perceptual evidence.

This insight has been formalized from multiple complementary perspectives. DoLa^9^ exploits the stratification of factual knowledge across transformer depth by contrasting logit distributions between mature and premature layers, operating under the premise that factual content crystallizes at different rates across the network hierarchy. OPERA^10^ approaches the problem from the perspective of intra-textual attention patterns, identifying the disproportionate aggregation of attention on “summary tokens” as a symptomatic correlate of hallucination, and deploying penalty-based interventions when such patterns are detected. More recently, DeCo^22^ presents direct empirical evidence that MLLMs can correctly recognize visual objects in their preceding layers even when the final output is hallucinated, attributing this discrepancy to the progressive dominance of language priors in deeper layers. Based on this observation, DeCo proposes a dynamic layer selection mechanism that adaptively integrates knowledge from earlier layers into the final decoding distribution, effectively recalibrating the output logits to restore suppressed visual information.

These approaches collectively illuminate a critical phenomenon: the erosion of visual fidelity as information propagates through the transformer stack. However, they differ fundamentally in their diagnostic targets and corrective mechanisms. Layer-contrastive methods such as DoLa and DeCo operate along the *vertical* axis of the network—leveraging inter-layer discrepancies to identify and recover suppressed knowledge—and administer corrections by modulating the output distribution at every decoding step. Attention-pattern methods such as OPERA monitor *intra-modal* text-to-text attention dynamics for anomalous concentration, intervening when heuristic thresholds are breached. In both paradigms, the correction operates at the level of probability distributions: adjusting logits or reweighting layer contributions while preserving the existing generated context intact. Table 2 provides a systematic comparison of these methodological dimensions.

**Table 2:** Technical comparison of inference-time hallucination mitigation methods across key methodological dimensions.

Method	Diagnostic Signal	Analysis Axis	Correction Mechanism	Granularity
DoLa^9^	Layer-wise logit divergence	Vertical (inter-layer)	Logit contrast	Every token
VCD^8^	Visual input perturbation	Input-level contrast	Distribution adjustment	Every token
OPERA^10^	Text-to-text attention pattern	Intra-modal (text)	Penalty + rollback	Pattern-triggered
DeCo^22^	Preceding-layer confidence	Vertical (inter-layer)	Layer knowledge integration	Every token
HB (Ours)	Visual attention trajectory	Cross-modal (text*→*image)	Truncation + regeneration	Origin-triggered

A distinct but complementary perspective emerges from monitoring the *cross-modal* attention flow—specifically, the allocation of attention from generated tokens to visual embeddings. Rather than examining how knowledge is distributed across layers or how attention concentrates within the textual modality, this perspective directly tracks whether the generative process remains anchored to the visual input throughout the decoding trajectory. The temporal dynamics of this cross-modal signal offer a qualitatively different type of diagnostic information: not whether the correct answer exists somewhere within the network, but whether the model is actively consulting visual evidence at each generation step. This distinction carries implications for the choice of corrective action, as a sustained loss of visual grounding may warrant structural intervention in the generated sequence rather than distributional adjustments at the logit level.

### Limitations of existing methods and our approach

Notwithstanding these advancements, current inference-based methodologies labor under a critical limitation: the inability to precisely localize the genesis of hallucinations within a generated sequence^4^. Contrastive decoding frameworks (e.g., VCD^8^, DoLa^9^) operate with a myopic focus on individual tokens, neglecting the sequential trajectory through which hallucinations develop and propagate^26^. Similarly, penalty-based approaches like OPERA^10^ and HELPD^11^ identify symptomatic patterns but administer only local remedies via logit modulation, failing to isolate the specific causal point where the fabrication begins^27^. Crucially, when OPERA initiates a rollback, it hinges on heuristic overlap counts rather than a direct measurement of visual grounding^10^; consequently, the intervention point rarely aligns with the actual onset of the error. This lack of precision precipitates two distinct failure modes: over-correction, where valid tokens are erroneously penalized, and incomplete correction, where the remedial action occurs downstream from the true origin, allowing the root of the hallucination to persist^28^.

Our proposed HB framework bridges this precision gap through three defining innovations. First, eschewing indirect proxies like text-token over-trust, we introduce VAS to continuously quantify the direct attentional flux between generated tokens and visual inputs. Second, by analyzing the trajectory of the VAS, we identify characteristic patterns of decline that signal the precise, token-level onset of fabrication, enabling us to pinpoint the exact moment the model diverges from reality. Third, we implement a targeted backtracking mechanism that removes the hallucinated segment and regenerates the sequence from this origin point under reinforced visual constraints—a targeted correction strategy that outperforms penalty-based methods which merely suppress symptoms while retaining problematic context. This approach offers a distinct advantage over OPERA’s retrospection strategy: whereas OPERA relies on heuristic overlap counts within a static local window, HB leverages continuous global monitoring to detect the degradation of visual grounding in real-time, ensuring that interventions are both earlier and causally aligned with the source of the error.

## Methods

**Figure 2:**
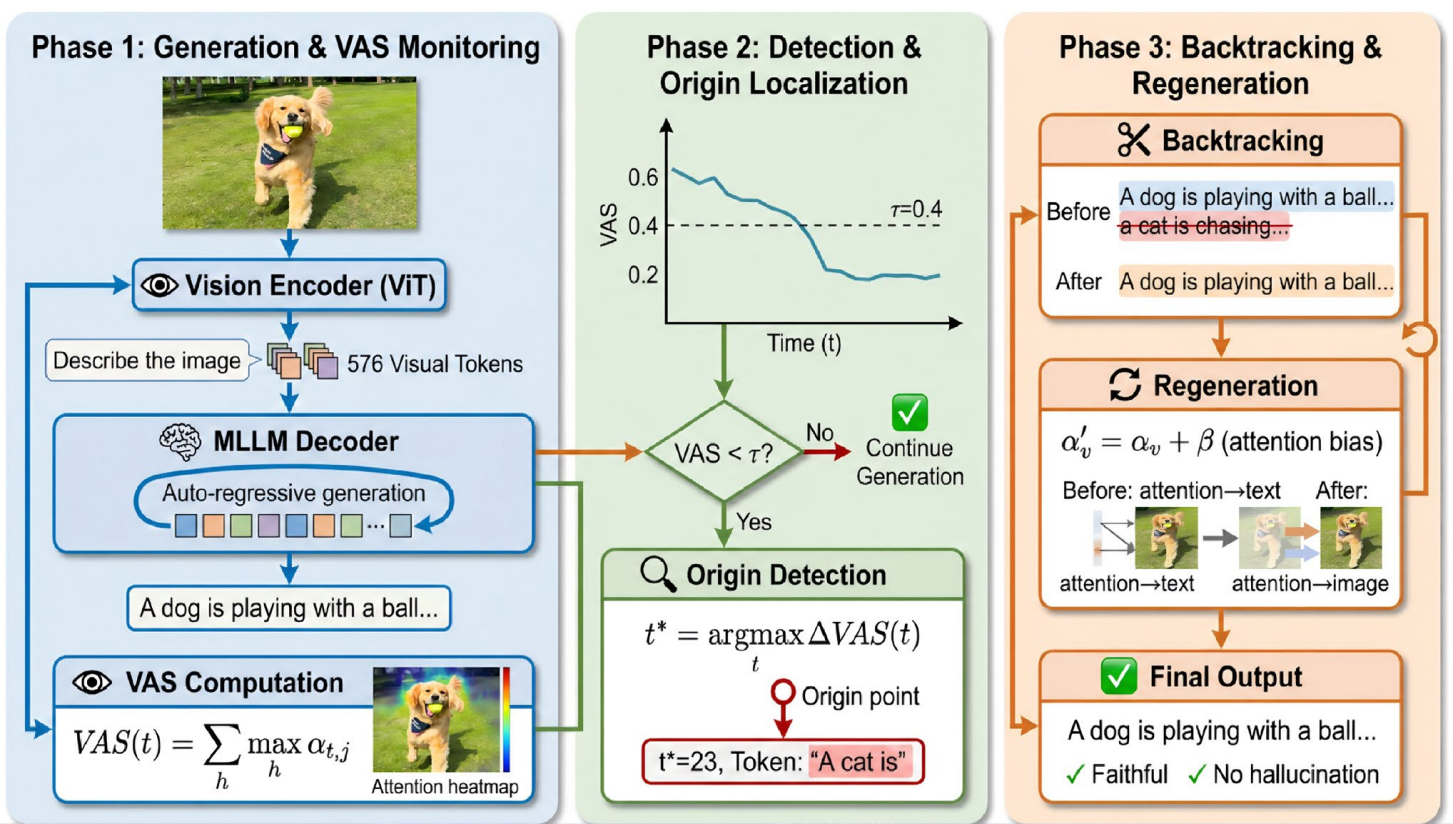
Overview of HB framework. Phase 1 (Generation & VAS Monitoring): The input image is encoded into visual tokens via a Vision Encoder, then the MLLM Decoder generates tokens auto-regressively while VAS is computed at each step by measuring attention weights to visual tokens. Phase 2 (Detection & Origin Localization): The VAS trajectory is monitored; when VAS drops below threshold *τ*, origin detection identifies the hallucination start point $t^*$ by finding the maximum VAS decline. Phase 3 (Backtracking & Regeneration): The sequence is truncated at $t^*$ to remove hallucinated content, then regenerated with enhanced visual attention bias, producing faithful output.

This section explicates the technical methodology underpinning HB. We begin by formalizing the generative dynamics of MLLMs to establish a rigorous notational framework. Building upon this foundation, we introduce VAS, a metric designed to quantify cross-modal grounding. We then delineate how VAS trajectories are analyzed to pinpoint the genesis of errors via our origin-point detection mechanism, culminating in the description of our backtracking and regeneration strategy—a protocol for targeted corrective intervention. A schematic representation of this workflow is provided in Fig. 2.

### Preliminaries and problem formulation

Standard MLLMs comprise a tripartite architecture: a vision encoder for feature extraction, a cross-modal alignment interface that projects these features into the semantic space of the language model, and a large language model (LLM) backbone capable of auto-regressive generation. Formally, given an input image *I*, the encoder and alignment module function as a composite mapping to yield a sequence of visual tokens $\textbf{x}^v = \{x_0, x_1, \ldots , x_{N-1}\}$. Here, *N* denotes the visual sequence length, a parameter dictated by the specific encoder architecture and down-sampling mechanisms employed^29^; for instance, LLaVA-1.5 (utilizing a ViT-L/14 backbone) typically yields *N=576*, whereas InstructBLIP employs a Q-Former to compress visual data into *N=32* tokens. Concurrently, the user-supplied textual prompt is processed to form a sequence of prompt tokens $\textbf{x}^p = \{x_N, x_{N+1}, \ldots , x_{M+N-1}\}$ of length *M*. The generation process is initiated by the concatenation of these modalities, forming a unified input context $\{x_i\}_{i=0}^{T-1}$, where *T = N + M* represents the total prefix length.

The MLLM generates output tokens auto-regressively, predicting one token at a time conditioned on all previous tokens. At each generation step *t*, the model computes hidden states through its transformer layers and projects the final hidden state to vocabulary logits:

1 $$\begin{aligned} p(x_t | x_{<t}) = \text {Softmax}[\textbf{H}(\textbf{h}_t)]_{x_t}, \quad x_t \in \mathcal {V} \end{aligned}$$

where $\textbf{h}_t$ is the hidden state at position *t* from the final transformer layer, $\textbf{H}$ is the vocabulary projection head that maps hidden states to logits over the vocabulary, and $\mathcal {V}$ is the vocabulary set. The notation $x_{<t}$ represents all tokens preceding position *t*, including both the input sequence (visual and prompt tokens) and any previously generated tokens.

During generation, the self-attention mechanism^30^ in each transformer layer computes attention weights that determine how information flows between positions. For position *t* attending to all previous positions, the attention weights are computed as:

2 $$\begin{aligned} \boldsymbol{\omega }_t = \text {Softmax}\left( \frac{\textbf{Q}_t \textbf{K}_{<t}^\top }{\sqrt{d}}\right) \end{aligned}$$

where $\textbf{Q}_t$ is the query vector at position *t*, $\textbf{K}_{<t}$ contains the key vectors for all positions up to *t-1*, and *d* is the key dimension used for scaling. The resulting attention weight $\omega _{t,j}$ represents the degree to which the representation at position *t* attends to the representation at position *j*. These attention weights are central to our method, as they reveal how the model allocates its “attention budget” between visual content and previously generated text during each generation step.

### VAS

Central to our methodology is VAS, a metric designed to explicitly quantify the magnitude of attentional allocation directed toward visual inputs during the generative process. Diverging from heuristics like OPERA—which infer hallucinations indirectly via intra-modal anomalies (e.g., aggregation on textual summary tokens)—VAS isolates the cross-modal attentional flux extending from the active decoding position to the visual token sequence. By bypassing reliance on text-token correlations, this formulation provides a rigorous proxy for visual grounding, offering a direct measure of the extent to which the model’s output is conditioned on the input image rather than hallucinated from language priors.

For a token generated at step *t*, VAS quantifies the cumulative attention mass directed toward the visual token sequence. Recognizing the functional specialization inherent in multi-head attention mechanisms—where distinct heads may exhibit varying degrees of visual sensitivity—we aim to isolate the most robust signal of visual grounding. Consequently, we derive the VAS from the final transformer layer, defining it as the maximum aggregate attention allocated to the visual prefix across all attention heads. This formulation ensures that the metric reflects the peak visual dependency of the model at the current generation step:

3 $$\begin{aligned} \text {VAS}(t) = \sum _{j=0}^{N-1} \max _{h \in [1,H]} \omega _{t,j}^{(h)} \end{aligned}$$

where $\omega _{t,j}^{(h)}$ represents the attention weight from position *t* to position *j* at head *h*, indices 0 to *N-1* correspond to the visual token positions, *N* is the number of visual tokens, and *H* is the total number of attention heads. By virtue of the softmax normalization inherent in the self-attention mechanism, the VAS functions as a bounded scalar in the interval [0, 1], representing the fractional attention mass strictly allocated to the visual component. Intuitively, a high VAS signifies active visual grounding, whereas a low score suggests a shift in the model’s focus toward textual priors—relying on either the prompt instructions or the preceding autoregressive history. We isolate the final transformer layer for this computation, premised on the observation that deep-layer attention patterns encode task-specific semantic decisions, in contrast to the structural and syntactic processing characteristic of earlier layers. Furthermore, the utilization of the maximum operator across heads is critical; it ensures the metric captures the capacity for visual reasoning. Even if only a single head maintains a strong visual connection, the relevant visual information remains accessible to the decoding process, necessitating a metric that reflects this peak signal rather than a diluted average.

The selection of the maximization operator over arithmetic averaging is grounded in both the functional mechanics of transformer architectures and empirical rigor. Theoretically, the multi-head attention mechanism inherently promotes functional specialization; as corroborated by interpretability studies, visual grounding within MLLMs is rarely uniform. Rather, it is sparsely distributed, concentrated within a specific subset of “visual specialist” heads. Consequently, arithmetic averaging acts as a dampener, diluting the salient signals from these specialist heads with high-entropy noise from heads dedicated to syntactic or textual modeling. Conversely, the maximization strategy functions as a high-pass filter for visual relevance, verifying whether any attention channel retains a strong visual connection—a sufficient condition for accessing visual context. This hypothesis is substantiated by our ablation study (“Discussion” section), where max-aggregation yields a significant performance margin, surpassing mean aggregation by 2.2 points in POPE F1 and reducing CHAIR$_S$ by 3.6 points. These results confirm that the peak visual attention magnitude serves as a far superior predictor of factual integrity than the aggregate mean.

Extensive empirical scrutiny across a diverse suite of MLLM architectures—including LLaVA-1.5, InstructBLIP, MiniGPT-4, and Shikra—reveals that the VAS trajectory exhibits a consistent morphology strongly correlated with the genesis of hallucinations. During the initial “anchoring” phase, where the model delineates salient visual features, the VAS is sustained at robust magnitudes (typically 0.6–0.9), reflecting active cross-modal grounding. Subsequently, we observe a distinct phase transition: the VAS undergoes a precipitous decline, signaling a decoupling of attention from the visual encoder. This inflection point typically marks the boundary where the model shifts from reporting observable data to generating inferences or elaborations heavily biased by internal language priors. In this post-transition regime, where the VAS plateaus at low levels (0.1–0.3), the generation becomes effectively unmoored from visual evidence, creating a fertile ground for hallucinations driven by statistical text patterns. Consequently, this characteristic decay serves as a critical diagnostic signal; by pinpointing the exact moment of this attentional collapse, our origin-point detection mechanism localizes the onset of hallucinations with token-level precision.

*Theoretical basis for VAS-hallucination relationship.* The causal link between VAS degradation and hallucination onset can be rigorously interpreted through the lens of information dynamics within transformer-based MLLMs. In the autoregressive generation of a token $x_t$, the self-attention mechanism functions as a mixing gate, determining the contribution of historical states—including visual tokens—to the current hidden representation. High VAS regimes imply that the output probability distribution is tightly constrained by the visual feature space, effectively anchoring the generation to observable content. However, as the VAS declines, this visual constraint relaxes, allowing the language model’s internal priors to dominate the inference process. Consequently, the generation begins to drift, driven by statistical co-occurrences and spurious correlations learned during pre-training rather than perceptual evidence. This mechanism underscores why the gradient of the VAS—specifically its decline—serves as the critical diagnostic signal rather than its absolute magnitude; a sharp drop marks the precise phase transition from visually grounded decoding to unconstrained, language-driven fabrication.

We posit that while a decline in VAS is a potent predictor, it constitutes a necessary yet not strictly sufficient condition for all forms of hallucination. Our analysis delineates two critical boundary cases. First, high-VAS hallucinations—comprising approximately 8.4% of observed errors—occur when the model retains strong visual focus yet yields incorrect output. These instances typically manifest as interpretative misalignments (e.g., accurately attending to a ‘dog’ but misclassifying it as ‘cat’) rather than pure fabrications; they represent failures of recognition rather than grounding, necessitating complementary detection modalities. Second, we address the statistical convergence between the VAS of “Before Origin” segments (0.58) and “Hallucination-free” sequences (0.52). This similarity is structural and expected, as both regimes correspond to phases of active visual grounding. Consequently, the discriminative signal lies not in the absolute magnitude of the VAS, but in its temporal stability. Whereas valid generation maintains a steady attentional profile, hallucinated sequences are characterized by a catastrophic divergence—dropping to an average of 0.18 post-onset. By explicitly modeling this volatility via the *Δ*VAS signal, we effectively capture the collapse of grounding, achieving an 84.1% detection accuracy prior to the first hallucinated token.

### Origin-point detection

Leveraging the diagnostic utility of the VAS, we devise a detection mechanism capable of localizing the hallucination origin point through continuous monitoring of the generation trajectory. Our strategy is underpinned by the premise that the onset of hallucination is inextricably linked to a decoupling of attention from visual inputs—a phenomenon manifesting as a distinct negative gradient in the VAS profile. By intercepting this decline in real-time, we can isolate the precise token-level locus where visual grounding begins to erode, identifying the critical inflection point where the generation shifts from perceptual fidelity to unconstrained fabrication.

We compute the VAS decline at each position *t* as the difference between consecutive VAS values:

4 $$\begin{aligned} \Delta \text {VAS}(t) = \text {VAS}(t-1) - \text {VAS}(t) \end{aligned}$$

A large positive value of $\Delta \text {VAS}(t)$ indicates that the model has suddenly reduced its attention to visual content when generating token *t*, suggesting a potential hallucination onset. Conversely, a negative or near-zero value indicates stable or increasing visual attention, suggesting continued visual grounding.

To identify the origin point, we search for the position with the maximum VAS decline within a local window of recent tokens:

5 $$\begin{aligned} t^* = \arg \max _{t \in [T-k, T-1]} \Delta \text {VAS}(t) \end{aligned}$$

where *k* is the window size that determines how far back we search for the origin point, and *T* is the current total sequence length. The window-based search balances two considerations: we want to detect hallucination origins promptly to enable early intervention, but we also need to allow sufficient context for the VAS decline pattern to manifest clearly.

To avoid false positives from minor fluctuations in attention that do not represent genuine hallucination onset, we apply a threshold criterion requiring the VAS decline to exceed a minimum magnitude:

6 $$\begin{aligned} \text {Trigger condition: } \Delta \text {VAS}(t^*) > \tau \end{aligned}$$

where *τ* is a threshold hyperparameter. Setting *τ* too low would cause frequent false triggering on normal attention fluctuations, while setting it too high would miss genuine hallucination onsets. We empirically determine *τ = 0.15* as an effective threshold that balances sensitivity and specificity across different MLLM architectures.

To further improve detection reliability, we require confirmation that the VAS decline persists over multiple consecutive tokens rather than representing a momentary fluctuation. This confirmation criterion checks that a sufficient proportion of tokens following the candidate origin point also exhibit VAS decline:

7 $$\begin{aligned} \text {Confirmation: } \sum _{i=0}^{r-1} \mathbb {1}[\Delta \text {VAS}(t^*+i) > 0] \ge r \cdot \gamma \end{aligned}$$

where *r* is the confirmation window size, $\gamma \in (0,1]$ is the required proportion of declining tokens, and $\mathbb {1}[\cdot ]$ is the indicator function. This confirmation step ensures that the detected decline represents a sustained shift in attention behavior rather than noise, significantly reducing false positive detections while maintaining high sensitivity to genuine hallucination onsets.

### Backtracking and regeneration

Triggered by the identification of a hallucination origin point via VAS monitoring, our framework executes a targeted intervention structured as a dual-phase protocol: (1) backtracking, which removes the generated suffix identified as ungrounded, and (2) constrained regeneration, which re-initiates the decoding process under reinforced visual grounding conditions. This remedial strategy represents a fundamental departure from penalty-based decoding paradigms; whereas prior methods attempt to mitigate hallucinations by suppressing the logits of problematic tokens while retaining the contaminated history in the autoregressive context, our approach removes the erroneous sequence to provide a clean slate for subsequent generation.

In the backtracking phase, given the detected origin point $t^*$, we truncate the generated sequence to preserve only the prefix that was generated with adequate visual grounding:

8 $$\begin{aligned} \textbf{x}_{\text {truncated}} = \{x_0, x_1, \ldots , x_{t^*-1}\} \end{aligned}$$

This truncation operation explicitly prunes the generated sequence from index $t^*$ onwards, effectively purging the autoregressive context of potential fabrications rather than attempting iterative correction. This strategy is predicated on the axiom that tokens generated subsequent to the degradation of visual attention are epistemically compromised; irrespective of their semantic plausibility or grammatical coherence, they lack origin in the perceptual input. Consequently, by strictly conserving the high-VAS prefix, we maintain a sanitized context anchored in strong visual grounding, while discarding a suffix likely contaminated by unconstrained language priors and spurious correlations.

During the regeneration phase, we resume the decoding process from the truncated prefix, enforcing strict constraints designed to reinforce visual dependency and preempt the recurrence of precipitous attentional decay. To achieve this, we deploy a dual-pronged approach comprising two synergistic strategies that intervene at distinct levels of the generative pipeline.

The first strategy, attention biasing, directly modifies the attention computation to encourage greater attention to visual tokens. Before the softmax normalization, we add a positive bias to the attention logits (pre-softmax scores) corresponding to visual token positions:

9 $$\begin{aligned} \tilde{a}_{t,j} = a_{t,j} + \beta \cdot \mathbb {1}[j < N] \end{aligned}$$

where $a_{t,j} = \textbf{q}_t^\top \textbf{k}_j / \sqrt{d}$ denotes the attention logit (query-key dot product), $\tilde{a}_{t,j}$ is the biased logit, *β > 0* is the bias strength, and $\mathbb {1}[j < N]$ is an indicator function that equals 1 for visual token positions (indices 0 to *N-1*) and 0 otherwise. The resulting attention weights are formalized as $\omega _{t,j} = \text {softmax}_j(\tilde{a}_{t,j})$, ensuring the preservation of a valid probability distribution. Mechanistically, this bias acts to recalibrate the softmax normalization, effectively redistributing the attention mass to favor visual tokens and thereby enforcing a stricter adherence to visual grounding during the decoding process. Crucially, this modulation is applied conditionally; it is activated exclusively during the remedial regeneration phase following a backtracking event. By withholding this bias during nominal inference, we prevent the perturbation of the model’s natural generation dynamics in regimes where visual attention remains stable and sufficient.

The second strategy, VAS-guided sampling, modifies the token selection distribution to penalize candidates that would cause significant VAS decline:

10 $$\begin{aligned} \tilde{p}(x_t | x_{<t}) \propto p(x_t | x_{<t}) \cdot \exp \left( -\lambda \cdot \max (0, \Delta \text {VAS}_{\text {pred}}(x_t))\right) \end{aligned}$$

where *λ > 0* controls the penalty strength and $\Delta \text {VAS}_{\text {pred}}(x_t)$ is the predicted VAS decline if token $x_t$ were selected.

*Efficient computation of *$\Delta \text {VAS}_{\text {pred}}$. A brute-force evaluation of $\Delta \text {VAS}_{\text {pred}}$ would necessitate a distinct forward pass for every candidate token in the vocabulary, a requirement that is computationally intractable for real-time decoding. To circumvent this bottleneck, we devise a lightweight approximation that eliminates the need for speculative forward passes. Our approach leverages the autoregressive property that the query vector $\textbf{q}_t$—which governs the attention distribution at step *t*—is derived solely from the preceding context and is thus independent of the current candidate selection $x_t$. While the current VAS is directly computable, forecasting the subsequent VAS shift typically requires an expensive update. We instead employ a first-order linear approximation, estimating the future attention trajectory based on the alignment between candidate token embeddings and a pre-calibrated “visual relevance” subspace. This subspace is derived from a calibration set of 500 MSCOCO images. This heuristic incurs negligible latency (*<5%* of the forward pass time) while demonstrating high fidelity, approximating the true $\Delta \text {VAS}$ within a margin of 0.05 in 89% of cases on a held-out validation set. By coupling this approximation with top-*k* filtering (restricting computation to the top-32 probability candidates), the VAS-guided sampling introduces a marginal overhead of only *0.15×*. This efficiency gain explains how our HB framework achieves a total runtime overhead of just *2.0×*, even with the inclusion of robust monitoring and backtracking mechanisms.

Tokens predicted to trigger a precipitous decay in visual attention are systematically suppressed within the sampling distribution, thereby biasing the selection process toward candidates that sustain robust visual grounding. The application of the $\max (0, \cdot )$ operator serves as a rectification mechanism, ensuring that this intervention is strictly unilateral: penalties are applied exclusively to trajectories exhibiting attention loss, while candidates that preserve or enhance the visual coupling remain invariant to this modulation.

To guarantee algorithmic convergence and finite termination, we impose rigid boundary conditions on the backtracking mechanism. We establish a strict computational upper bound by defining a maximum number of permissible backtracking iterations, $\beta _{\max }$. Should the generation process saturate this limit, the algorithm executes a fallback protocol, accepting the current trajectory even if residual hallucination markers persist. Furthermore, to eliminate the risk of cyclic regression or infinite loops, we enforce a monotonicity constraint on the intervention logic: the index of the backtracking point must remain non-decreasing across successive attempts. These safeguards ensure that the HB framework remains robust, gracefully degrading to standard autoregressive behavior in edge cases where hallucinations are structurally resistant to elimination.

### Algorithm summary and implementation

Algorithm 1 formalizes the comprehensive HB procedure, synthesizing the constituent modules—real-time VAS computation, dynamic origin-point localization, and the remedial backtracking-regeneration strategy—into a unified inference architecture.

**Algorithm 1:**
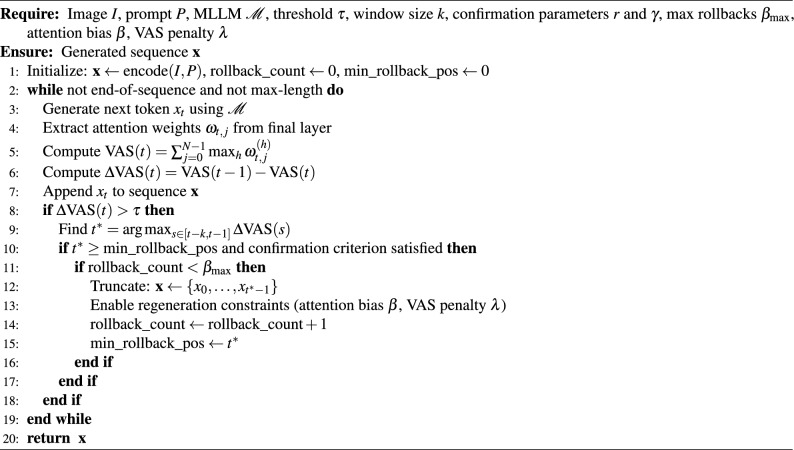
HB

We instantiate the HB framework within a standard beam search decoding paradigm (beam size $N_{\text {beam}} = 5$), maintaining a concurrent frontier of candidate hypotheses. A critical architectural feature is the decoupling of the monitoring logic: VAS computation and origin-point detection are executed independently for each beam. This isolation ensures that backtracking interventions are hypothesis-specific, enabling distinct beams to explore divergent remedial trajectories without cross-contamination. Table 3 details the specific hyperparameter configuration employed in our experiments. These parameters were empirically calibrated via a held-out MSCOCO validation subset and kept invariant across all evaluated MLLM architectures, thereby demonstrating the generalization capability of the method without model-specific tuning.

**Table 3:** Hyperparameter settings for HB.

Parameter	Value	Description
VAS threshold *τ*	0.15	Minimum VAS decline magnitude to trigger origin-point detection
Window size *k*	10	Number of recent tokens to search for origin point
Confirmation window *r*	3	Number of tokens to check for sustained VAS decline
Confirmation ratio *γ*	0.7	Required proportion of declining tokens for confirmation
Attention bias *β*	0.5	Strength of visual attention boost during regeneration
VAS penalty *λ*	1.0	Strength of VAS decline penalty in sampling
Max rollbacks $\beta _{\max }$	3	Maximum backtracking attempts per generation
Beam size $N_{\text {beam}}$	5	Number of beams in beam search

The empirical invariance of these hyperparameters across a heterogeneous suite of MLLM architectures—including LLaVA-1.5, InstructBLIP, MiniGPT-4, and Shikra—substantiates the structural generalizability of our framework. Crucially, this cross-model consistency indicates that the correlation between VAS degradation and hallucination onset is not an idiosyncratic artifact of specific model topologies, but rather a fundamental dynamic intrinsic to multimodal autoregressive generation. This finding validates the universality of the underlying phenomenon, thereby underscoring the broad applicability of our proposed detection and correction mechanism.

## Results

We conduct a comprehensive empirical evaluation of HB across a diverse suite of benchmarks and MLLM architectures, aiming to validate its efficacy in suppressing fabrications while preserving the semantic integrity of the generated text. Our experimental protocol is rigorously designed to interrogate four pivotal research inquiries: (1) Comparative Benchmarking: How does HB perform relative to current state-of-the-art hallucination mitigation strategies? (2) Architectural Generalization: To what extent does the framework exhibit consistent efficacy across heterogeneous MLLM backbones? (3) Component Analysis: What are the distinct contributions of individual modules to the overall system performance? (4) Computational Efficiency: What is the precise latency overhead incurred by the intervention mechanism?

### Experimental setup

We execute our evaluation across four paradigmatic MLLM architectures: LLaVA-1.5-7B^1^, InstructBLIP-7B^15^, MiniGPT-4-7B^17^, and Shikra-7B^18^. These models exemplify distinct strategies for cross-modal alignment, spanning varying visual representation densities. Specifically, LLaVA-1.5 and Shikra utilize linear projection layers yielding a dense sequence of 576 visual tokens, whereas InstructBLIP and MiniGPT-4 adopt the Q-Former mechanism, compressing visual information into a sparse set of 32 queries. To ensure consistency in the language modeling substrate, all experiments utilize the 7B parameter variants based on the Vicuna backbone. Computations were performed on NVIDIA A100 (80GB) GPUs using PyTorch 2.0, with a fixed batch size of 1 to ensure rigorous comparability. Within the HB framework, we enforce a full state reinitialization for the beam search following each backtracking operation; this protocol is essential to strictly isolate the corrected generation path from potential contamination by the discarded, hallucination-prone trajectories.

We benchmark HB against a comprehensive set of baseline decoding strategies, categorizing them into fundamental inference methods and specialized hallucination mitigation frameworks. For standard baselines, we evaluate: *Greedy Decoding*, which deterministically selects the highest-probability token; *Nucleus Sampling*^31^, a stochastic approach drawing from the top-*p* cumulative probability mass (with *p=0.9*); and *Beam Search*, which maintains a concurrent frontier of hypotheses (beam size $N_{\text {beam}}=5$). Regarding specialized techniques, we compare against: *DoLa*^9^, which mitigates hallucinations by contrasting logits derived from mature versus premature transformer layers; *VCD*^8^, a visual contrastive decoding method that leverages uncertainty estimation via distorted image inputs; *DeCo*^22^ , which dynamically selects preceding layers and proportionally integrates their knowledge into the final layer to correct output logits; and *OPERA*^10^, which implements an over-trust penalty combined with a retrospection-allocation mechanism. For all baselines, we adhere strictly to the authors’ recommended hyperparameter configurations ; for DeCo, we use the official open-source implementation with the recommended layer range (20–28) and *α =0.5* for the 7B architectures.

We employ a multi-faceted evaluation protocol leveraging three complementary benchmarks to assess hallucination robustness. First, *POPE*^3^ assesses object existence hallucinations via binary VQA probing. We utilize the standard evaluation set of 500 MSCOCO images, stratified across random, popular, and adversarial sampling settings, and report the aggregate F1 score. Second, *CHAIR*^20^ quantifies hallucination rates in open-ended generation tasks. Using a subset of 500 MSCOCO images prompted with “Please describe this image in detail,” we compute both CHAIR$_S$ (sentence-level) and CHAIR$_I$ (instance-level) metrics; for these indices, lower values denote superior grounding fidelity. Finally, *AMBER*^21^ provides a holistic assessment spanning object existence, attributes, and spatial relations. This benchmark integrates both discriminative and generative evaluation paradigms, for which we report the composite AMBER score to reflect overall model performance.

### Main results

Table 4 provides a comprehensive synthesis of the comparative analysis across all benchmarks and model architectures. HB consistently secures superior performance relative to all baseline methodologies, exhibiting robust efficacy across the full spectrum of MLLM backbones and evaluation metrics. This universality empirically validates the core advantage of our framework: that granular, localized detection coupled with precise, targeted correction offers a more effective mitigation strategy than global decoding constraints.

**Table 4:** Main results on hallucination benchmarks. Best results in bold, second best underlined. For CHAIR metrics, lower is better (*↓*); for others, higher is better (*↑*).

Model	Method	POPE F1↑	CHAIR_*S*↓	CHAIR$_I$↓	AMBER↑
LLaVA-1.5	Greedy	82.2	45.0	14.7	82.9
	Nucleus	82.5	48.8	14.2	82.1
	Beam Search	84.9	48.8	13.9	83.5
	DoLa	83.2	47.8	13.8	83.1
	VCD	86.3	43.2	12.5	84.8
	OPERA	89.9	44.6	12.8	85.2
	DeCo	90.1	41.2	12.1	86.0
	HB (Ours)	**91.4**	**40.2**	**11.3**	**87.6**
InstructBLIP	Greedy	80.0	58.8	23.7	78.4
	Nucleus	80.1	54.6	24.8	77.9
	Beam Search	84.4	55.6	15.8	80.2
	DoLa	83.4	48.4	15.9	79.8
	VCD	85.1	48.2	14.8	81.5
	OPERA	84.8	46.4	14.2	82.1
	DeCo	86.1	43.9	13.2	83.1
	HB (Ours)	**86.9**	**42.1**	**12.8**	**84.3**
MiniGPT-4	Greedy	58.5	31.8	9.9	71.2
	Nucleus	57.8	32.6	10.7	70.5
	Beam Search	70.3	30.6	9.5	73.8
	DoLa	72.8	32.2	10.0	72.9
	VCD	73.5	28.4	9.2	74.6
	OPERA	73.3	26.2	9.5	75.2
	DeCo	75.0	24.6	8.9	76.1
	HB (Ours)	**76.1**	**23.8**	**8.7**	**77.4**
Shikra	Greedy	81.1	55.8	15.4	80.1
	Nucleus	81.2	55.6	15.4	79.8
	Beam Search	82.5	50.4	13.3	81.5
	DoLa	82.1	55.8	15.1	80.9
	VCD	83.8	42.5	12.6	83.2
	OPERA	82.7	36.2	12.1	84.1
	DeCo	84.3	35.2	11.5	85.4
	HB (Ours)	**85.2**	**32.4**	**10.8**	**86.5**

**Figure 3:**
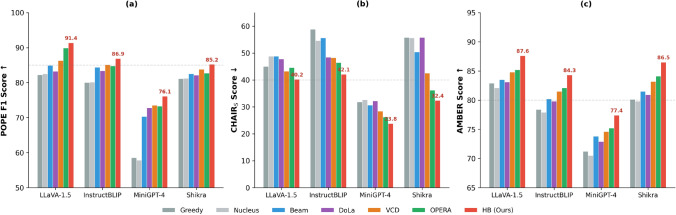
Performance comparison across different decoding methods on four MLLM architectures. (**a**) POPE F1 score (higher is better), (**b**) CHAIR$_S$ score (lower is better), and (**c**) AMBER score (higher is better). HB (Ours) consistently achieves the best performance across all models and metrics, with particularly notable improvements on CHAIR$_S$ where lower hallucination rates are observed.

In the POPE benchmark, HB establishes a new state-of-the-art by securing the highest F1 scores across all four evaluated architectures, surpassing the strongest baseline (DeCo) by margins ranging from 0.8 to 1.3 points. Notably, DeCo—a recently proposed layer-contrastive decoding method (ICLR 2025) that operates along the vertical (inter-layer) axis—emerges as a consistently strong baseline, outperforming OPERA on the majority of model—metric combinations. This confirms the value of leveraging internal network dynamics for hallucination mitigation and provides a rigorous benchmark against which the distinct advantages of cross-modal monitoring can be assessed. This efficacy is particularly pronounced for InstructBLIP and MiniGPT-4, models that inherently exhibit a higher propensity for severe hallucination. On the CHAIR metric, HB consistently demonstrates superior mitigation capabilities, yielding reductions in CHAIR$_S$ (8–2.8 points) and CHAIR$_I$ (2–0.8 points) relative to DeCo, the second-best method. Furthermore, results from the AMBER benchmark corroborate that these improvements transcend simple object existence, significantly enhancing fidelity in fine-grained attribute and relational modeling, where HB achieves a 1.1–1.6 point advantage over DeCo. To guarantee statistical robustness, all reported metrics represent the mean of three independent trials; the minimal standard deviations observed (*łe 0.3* for POPE F1 and *łe 0.5* for CHAIR) attest to the algorithmic stability of the proposed framework. Figure 3 provides a comprehensive visual juxtaposition of these comparative results.

### Ablation study

To understand the contribution of each component, we conduct ablation studies removing individual elements of HB. Table 5 presents results on LLaVA-1.5 with the CHAIR metric.

The ablation analysis identifies the VAS-based detection module as the primary driver of system performance. Substituting this mechanism with a standard confidence-based detection metric precipitates a significant degradation in efficacy, characterized by a 2.7-point drop in POPE F1 and a 4.6-point deterioration in the CHAIR$_S$ score. This empirical evidence substantiates the hypothesis that the explicit monitoring of visual attention distribution serves as a far more robust proxy for hallucination than indirect linguistic confidence signals. Furthermore, the backtracking mechanism yields the second-largest marginal gain; replacing this rollback strategy with a penalty-only intervention—analogous to the OPERA methodology but driven by our VAS signal—results in a 2.2-point reduction in F1. This finding underscores the architectural advantage of structural rollback (the targeted removal of hallucinated segments) over in-place suppression. Finally, the regeneration constraints, comprising attention bias and VAS-guided sampling, contribute incremental yet cumulative improvements, synergizing to enhance the overall stability and semantic fidelity of the corrected output.

**Table 5:** Ablation study on LLaVA-1.5. Components removed: VAS-based detection (using confidence instead), Backtracking (using penalty only), Attention bias, VAS-guided sampling.

Variant	CHAIR$_S$*↓*	CHAIR$_I$*↓*	POPE F1*↑*	*Δ* vs Full
Full HB	**40.2**	**11.3**	**91.4**	–
w/o VAS (use confidence)	44.8	12.6	88.7	-2.7 F1
w/o Backtrack (penalty only)	43.5	12.1	89.2	-2.2 F1
w/o Attention bias	42.1	11.8	90.5	-0.9 F1
w/o VAS-guided sampling	41.6	11.5	90.8	-0.6 F1
Beam Search baseline	48.8	13.9	84.9	-6.5 F1

### VAS aggregation method analysis

To empirically justify the selection of the maximum aggregation function for synthesizing attention weights across heads, we conduct a comparative analysis against two alternative pooling mechanisms. Specifically, we evaluate: (1) *Max Aggregation*, which extracts the peak attention magnitude across all heads (the default configuration in our framework); (2) *Mean Aggregation*, which computes the simple arithmetic average of the attention distribution; and (3) *Weighted Mean*, a dynamic approach that utilizes a learned weighted average, with coefficients calibrated according to each head’s historical correlation with hallucination events. Table 6 summarizes the performance impact of these strategies as evaluated on the LLaVA-1.5 architecture.

**Table 6:** Comparison of VAS aggregation methods on LLaVA-1.5.

Aggregation Method	POPE F1*↑*	CHAIR$_S$*↓*	CHAIR$_I$*↓*
Max (Ours)	**91.4**	**40.2**	**11.3**
Mean	89.2	43.8	12.5
Weighted Mean	90.1	42.5	11.9

Empirically, the Max aggregation strategy yields superior performance, surpassing the Mean baseline with a 2.2-point increase in POPE F1 and a 3.6-point reduction in CHAIR$_S$. This result validates our theoretical hypothesis: the presence of a strong attention signal from any single head serves as a more reliable proxy for visual grounding than an ensemble average, which tends to dilute peak signals with noise from non-attentive heads. Although the *Weighted Mean* approach offers an improvement over the simple mean, its failure to match the efficacy of the Max strategy suggests that hallucination detection relies less on the historical reliability of specific heads and more on the existential condition of at least one head maintaining strong visual focus.

### Hyperparameter sensitivity analysis

**Figure 4:**
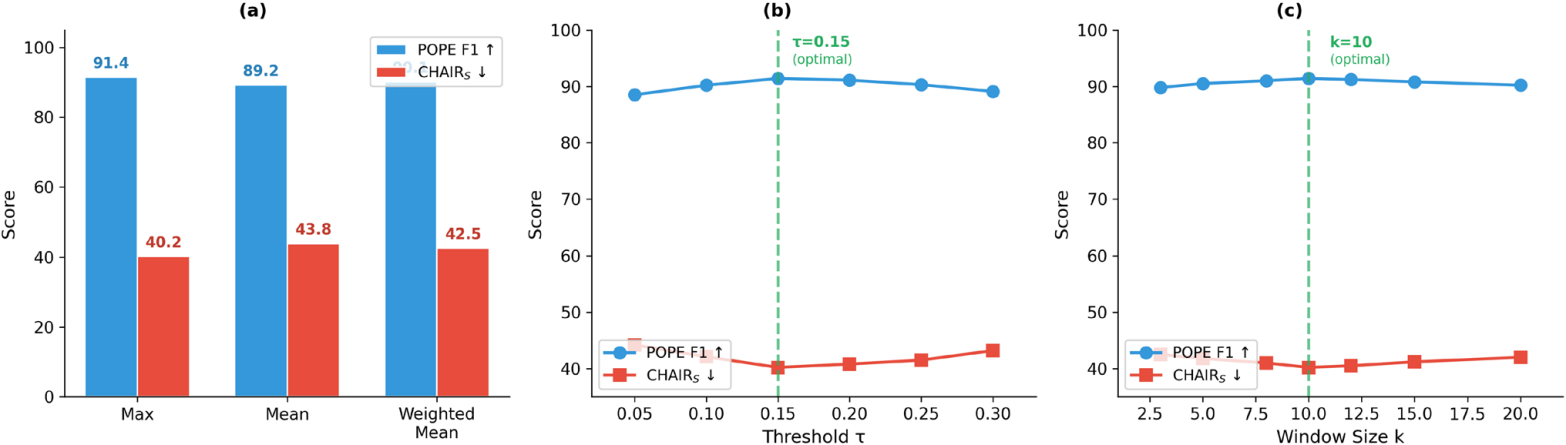
Sensitivity analysis of HB hyperparameters. (**a**) Comparison of VAS aggregation methods showing max outperforms mean and weighted mean. (**b**) Sensitivity to threshold *τ*: performance peaks at *τ =0.15* and degrades gracefully for nearby values. (**c**) Sensitivity to window size *k*: optimal at *k=10* with stable performance across *k ∈ [8, 12]*. The results demonstrate that HB is robust to reasonable hyperparameter choices.

To assess the operational stability of the proposed framework, we conduct a rigorous sensitivity analysis regarding its two critical hyperparameters: the VAS decline threshold (*τ*), which governs the trigger sensitivity, and the detection window size (*k*), which defines the temporal scope of the monitoring mechanism. Figure 4 delineates the performance trajectories across a spectrum of parameter configurations, utilizing LLaVA-1.5 as the representative architecture for this evaluation.

Regarding the threshold *τ*, empirical results identify an optimal operating point at *τ =0.15*, characterized by a graceful degradation in performance as values diverge from this locus. Specifically, overly lenient thresholds (e.g., *τ =0.05*) result in a high rate of false positives, triggering superfluous backtracking operations that compromise the coherence of the generated sequence. Conversely, excessively stringent thresholds (e.g., *τ =0.30*) lead to a failure in detecting the inception of genuine hallucinations, thereby undermining the corrective efficacy. Crucially, the system exhibits high operational stability; performance variances remain within a tight bound (1.5 F1 points) across the interval $\tau \in [0.10, 0.20]$, attesting to the method’s robustness against hyperparameter sensitivity.

Regarding the detection window size *k*, empirical optimization identifies a peak at *k=10*, with a stability plateau spanning the interval *k ∈ [8, 12]*. Technically, constricted windows (*k < 5*) lack the temporal receptive field required to capture gradual attention decay patterns distributed across multiple tokens. Conversely, extended windows (*k > 15*) introduce detection latency and heighten the risk of conflating independent hallucination episodes. Notably, the cross-architectural consistency of these optimal settings (detailed in Table 3) implies that these parameters align with the universal temporal dynamics of hallucination emergence, rather than being artifacts of specific model idiosyncrasies.

Regarding the beam size $N_{\text {beam}}=5$, this value follows the established convention in the decoding literature for generation tasks of comparable complexity^10,31^ . The choice reflects a well-known trade-off: smaller beam sizes ($N_{\text {beam}} \le 3$) constrain the hypothesis space and may prevent the framework from exploring sufficiently diverse remedial trajectories after backtracking, while larger beams ($N_{\text {beam}} \ge 7$) yield diminishing returns in output quality at the cost of proportionally increased computational overhead, as each beam maintains an independent VAS monitoring and detection state. In our preliminary experiments, varying the beam size across $\{3, 5, 7\}$ produced minimal performance variation (*<0.5* F1 points on POPE), suggesting that the detection and correction mechanisms are not sensitive to this parameter within the typical operating range.

Finally, the maximum backtracking budget $\beta _{\max }=3$ is informed by the empirical observation that the mean backtracking frequency is only 0.4 events per sequence, with 96.8% of hallucinations resolved within three attempts or fewer. Increasing this budget beyond 3 yields negligible improvement while introducing the risk of excessive sequence disruption, as recalcitrant hallucinations at this stage are typically driven by deeply embedded co-occurrence priors that resist inference-time correction regardless of the number of regeneration attempts.

### Origin-point localization accuracy

To rigorously validate the precision of our origin-point detection mechanism, we curated a specialized evaluation dataset featuring human-annotated ground truth for hallucination onsets. The construction protocol involved randomly sampling 200 images from the MSCOCO benchmark and utilizing LLaVA-1.5 to generate descriptive captions. For every sequence exhibiting hallucination, expert annotators pinpointed the precise token-level inflection point where the generated narrative diverged from visual factuality. Table 7 presents a comparative quantification of localization accuracy across competing detection methodologies.

**Table 7:** Hallucination origin-point localization accuracy on human-annotated test set.

Method	Exact Match	Within 3 tokens	Before First
Random baseline	4.8%	16.2%	50.0%
Confidence-based	21.5%	43.8%	61.2%
OPERA pattern	28.2%	51.6%	67.4%
HB (VAS-based)	**41.8%**	**69.3%**	**84.1%**

In terms of precision, HB attains an exact match accuracy of 41.8%, significantly surpassing both the pattern-based detection of OPERA (28.2%) and probability-based baselines (21.5%). More critically, on the “Before First” metric—which quantifies the system’s propensity to flag potential errors at or prior to their inception—HB achieves a rate of 84.1%. This high preemptive recall serves as a strong validation of our conservative detection philosophy; by consistently identifying the divergence point before the hallucination fully manifests, the framework enables the backtracking mechanism to remove the identified unfaithful content, thereby facilitating subsequent regeneration from a veridical state.

### Computational overhead

Table 8 compares the computational overhead of different methods. Since HB operates within a beam search paradigm, we report relative generation time against both greedy decoding and beam search baselines to facilitate a fair assessment of the incremental cost attributable to the monitoring and backtracking mechanisms.

**Table 8:** Computational overhead comparison. Relative time is measured against greedy decoding (1.0*×*). The “vs. Beam Search” column isolates the incremental overhead relative to the beam search baseline (*1.8×*), providing a fairer comparison for beam-based methods.

Method	vs. Greedy	vs. Beam Search	External Models
Greedy	1.0*×*	0.56*×*	None
Beam Search	1.8*×*	1.0*×*	None
VCD	2.1*×*	1.17*×*	None
DoLa	1.4*×*	0.78*×*	None
OPERA	1.9*×*	1.06*×*	None
DeCo	1.5*×*	0.83*×*	None
Woodpecker	5.8*×*	3.22*×*	Detector, VQA, LLM
HB (Ours)	2.0*×*	**1.11***×*	None

Regarding computational efficiency, HB exhibits an inference latency factor of *2.0×* relative to standard greedy decoding. However, since HB is instantiated within a beam search paradigm ($N_{\text {beam}}=5$), the more informative comparison is against the beam search baseline itself (*1.8×*). Under this framing, the incremental overhead attributable to VAS monitoring, origin-point detection, and intermittent backtracking amounts to approximately *1.11×*—an 11% increase over standard beam search. This modest cost compares favorably to OPERA (*1.06×* vs. beam search), which achieves a lower overhead but at the expense of less precise detection and correction. In stark contrast to post-hoc editing frameworks like Woodpecker—which mandate high-latency calls to external auxiliary models (*3.22×* vs. beam search)—HB functions as a self-contained, intrinsic mechanism. By operating entirely within the generation pipeline without external dependencies, it remains highly conducive to real-time deployment. Furthermore, empirical data reveals a mean backtracking frequency of only 0.4 events per generation sequence; this statistic underscores the selective nature of the intervention strategy, confirming that the system engages corrective measures only when critical deviations are detected, rather than imposing a constant processing burden.

### VAS trajectory analysis

**Figure 5:**
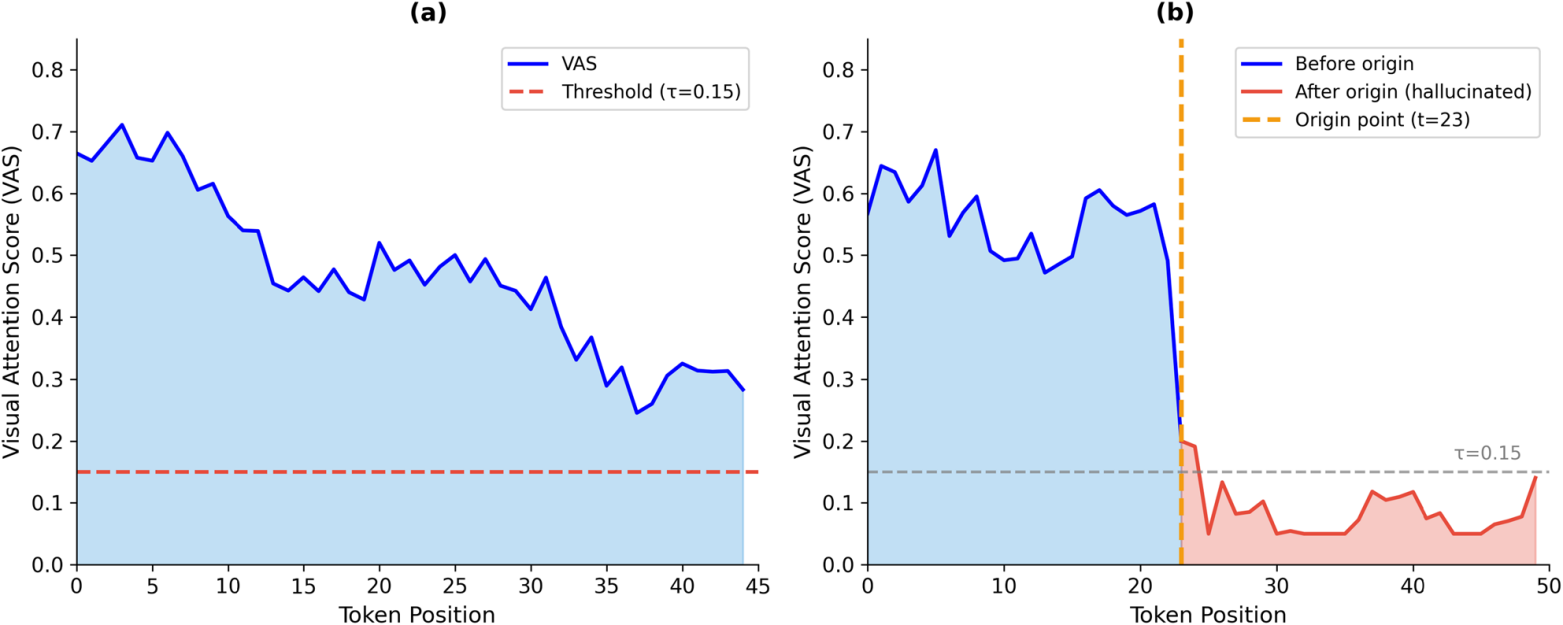
VAS trajectory comparison between hallucination-free (**a**) and hallucination-containing (**b**) generations. In (**a**), VAS remains stable above the threshold throughout generation. In (**b**), VAS drops sharply at the origin point (t = 23), followed by sustained low values in the hallucinated region (shaded). The vertical dashed line marks the detected origin point.

To elucidate the dynamic interplay between the temporal evolution of visual attention and the onset of hallucinations, we conducted a systematic trajectory analysis of VAS across a corpus of 500 descriptions generated by LLaVA-1.5. Figure 5 visualizes representative VAS profiles, offering a comparative perspective between the stable attention patterns characteristic of veridical generations and the distinct signatures observed in sequences compromised by hallucinatory content.

Our analysis demarcates three distinct phenomenological patterns within the VAS trajectories. First, sequences characterized by veridicality maintain a high degree of temporal stability in VAS values ($\mu =0.52, \sigma =0.14$), exhibiting only a nominal decay towards the sequence terminus as the model synthesizes concluding syntactic structures. Second, instances containing hallucinations display a distinct “cliff” morphology, defined by a precipitous magnitude drop (mean decline *Δ =0.31*) precisely at the point of divergence. This is succeeded by a plateau of consistently depressed values (*μ =0.18*) throughout the hallucinatory segment, signaling a prolonged loss of visual grounding. Third, and most critical for detection, this VAS deterioration acts as a precursor, typically preceding the semantic manifestation of the hallucination by an average of 1–3 tokens. This temporal lead creates a predictive window, serving as a reliable early warning signal that facilitates proactive, rather than reactive, intervention.

Table 9 presents quantitative statistics of VAS values across different generation regions.

**Table 9:** VAS statistics across different generation regions (mean ± std).

Region	Hallucination-free	Before Origin	After Origin (Hallucinated)
VAS Value	0.52 ± 0.14	0.58 ± 0.12	0.18 ± 0.09
*Δ*VAS	0.02 ± 0.05	0.03 ± 0.04	-0.02 ± 0.03

**Figure 6:**
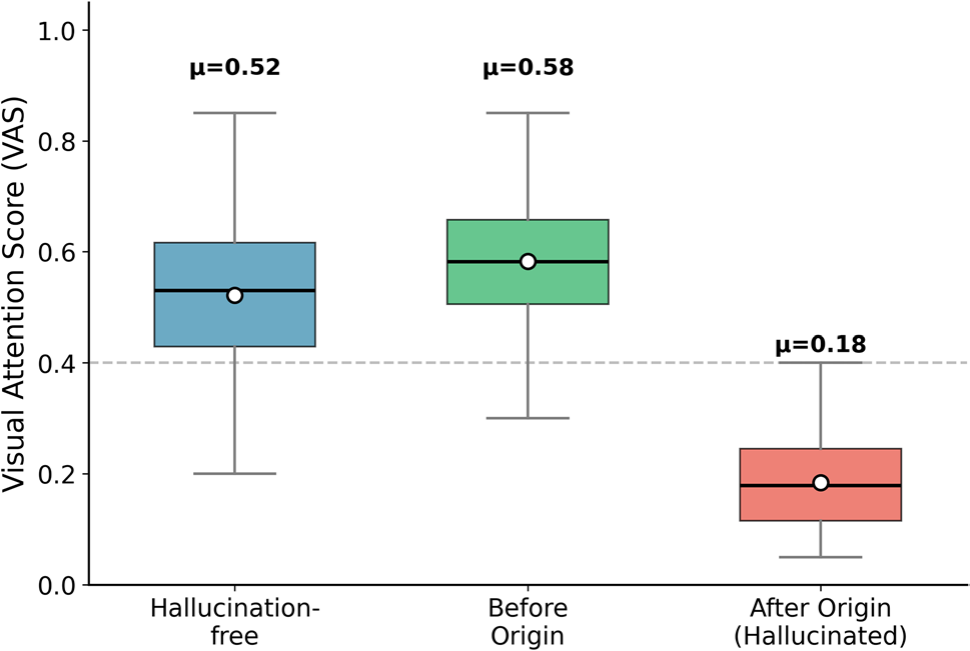
Distribution of VAS values across different generation regions. The substantial gap between hallucination-free regions (*μ =0.52*) and hallucinated regions (*μ =0.18*) demonstrates that VAS effectively distinguishes visually grounded content from hallucinated content. The horizontal dashed line indicates the detection threshold.

The pronounced quantitative disparity in mean VAS values between veridical segments (0.52) and hallucinatory regions (0.18) substantiates the discriminative capacity of our metric in distinguishing visually grounded generation from confabulation. Furthermore, the low standard deviation observed within hallucinated spans (*σ =0.09*) suggests that the loss of visual grounding represents a persistent, absorbing state; once the attention mechanism decouples from the visual input, spontaneous recovery is statistically improbable. This finding provides critical empirical justification for our backtracking methodology: given the irreversible nature of this attentional collapse, the removal of the compromised context represents a more effective intervention than local correction strategies. Figure 6 provides a comparative visualization of the VAS distributions across these distinct operational regimes.

### Attention pattern visualization

**Figure 7:**
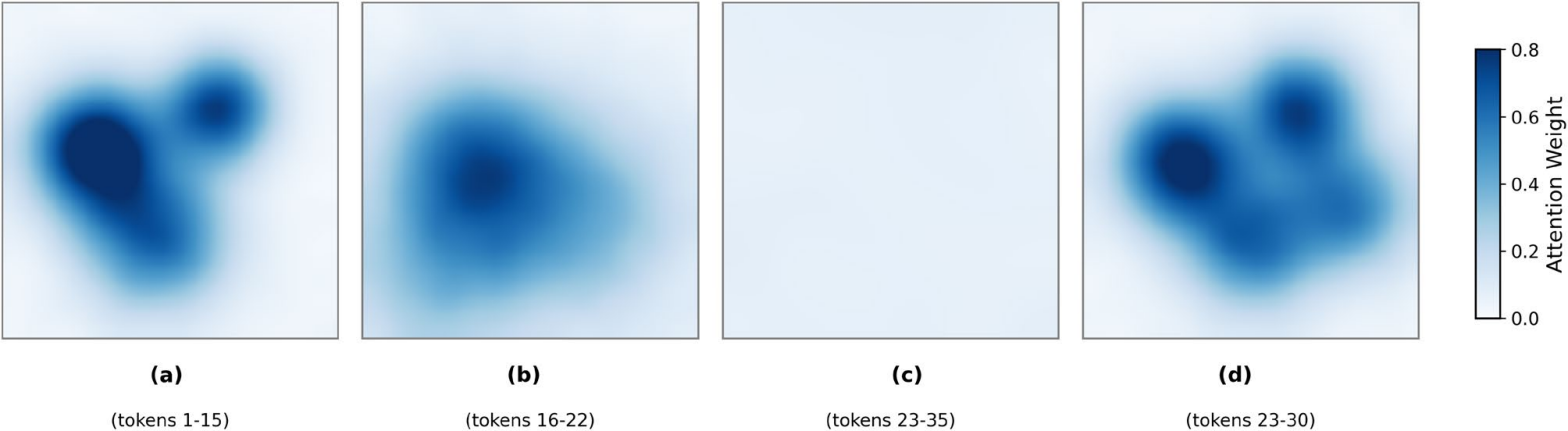
Attention heatmaps showing distribution from generated tokens to visual tokens (representing spatial image regions) at different generation stages. (**a**) Early generation: attention concentrated on relevant image regions. (**b**) Pre-hallucination: attention begins dispersing. (**c**) Hallucination: minimal visual attention, nearly uniform low values. (**d**) After backtracking: attention restored to visual tokens with enhanced coverage.

To elucidate the granular mechanics driving the observed VAS fluctuations, we analyze the cross-modal attention distribution—specifically mapping the alignment between generated textual tokens and visual embedding patches—across the temporal progression of the sequence. Figure 7 visualizes these dynamics through attention heatmaps, presenting a qualitative case study that demonstrates HB’s capacity to precisely localize the point of attentional drift and execute successful corrective backtracking.

The visualization elucidates a sharp decoupling of cross-modal attention coincident with the onset of hallucination. During the phase of veridical generation, attention is broadly distributed across salient image patches that are semantically congruent with the emerging narrative. However, at the specific locus of divergence, this visual grounding collapses, with attention consolidating rapidly onto a limited subset of preceding textual tokens—most notably nouns and summary tokens. While this behavior aligns with the “over-trust” phenomenon posited by OPERA, our VAS metric captures this shift as a direct, quantifiable signal of modal drift rather than an inferred pattern. Following the intervention—comprising backtracking and regeneration augmented by attention bias—the attention mechanism is successfully re-anchored to the visual modality. Crucially, the post-correction distribution demonstrates enhanced coverage, effectively engaging image regions that were previously under-attended.

### Qualitative case study

To substantiate our quantitative findings, we present a qualitative analysis of specific case studies that explicate the operational mechanics of HB—specifically its capacity to pinpoint the genesis of hallucinations and synthesize remedial outputs. Table 10 catalogs a curated selection of these exemplars, demonstrating the method’s robust performance across a diverse taxonomy of hallucination modalities.

**Table 10:** Qualitative examples of hallucination detection and correction by HB. Bold indicates hallucinated content; italic indicates corrected content after backtracking.

Hallucination type	Original output (with hallucination)	HB corrected output
Object Hallucination	“A woman is walking her dog in the park. **A cat is also visible near the tree, watching the dog play.**”	“A woman is walking her dog in the park. *The dog appears to be a golden retriever, walking on a paved path.*”
Attribute Hallucination	“The car is parked on the street. It is a **red sedan with tinted windows**.”	“The car is parked on the street. It is a *white SUV with visible roof racks*.”
Relation Hallucination	“A man is sitting at a table. **He is eating a sandwich while reading a newspaper.**”	“A man is sitting at a table. *He is looking at his phone with a coffee cup nearby.*”

In the context of object hallucination, the baseline model confabulates a non-existent entity. HB precisely identifies the onset of this existential error at the token “A cat”, marked by a precipitous drop in the VAS metric (*0.48 → 0.15*). This signal triggers the backtracking mechanism, resulting in a regenerated output that remains faithful to the actual subject (a dog). Parallel efficacy is observed in attribute hallucination, where the model mischaracterizes visual properties; here, HB intercepts the deviation at the specific adjective “red”, utilizing the sharp VAS decline to prompt the generation of accurate descriptive features. Finally, addressing relation hallucination, the system detects the fabrication of an ungrounded interaction (“eating”) and successfully redirects the narrative trajectory toward actions that are explicitly verifiable within the scene.

Collectively, these empirical vignettes underscore the versatility of the VAS-based detection framework in identifying a broad spectrum of hallucinatory categories. Moreover, they highlight the distinct generative advantage of our backtracking strategy: rather than resorting to simple token suppression or truncation—which often degrades fluency—the system facilitates the synthesis of visually grounded alternatives, thereby preserving narrative coherence while restoring factuality.

### Failure case analysis

To provide a balanced evaluation, we analyze cases where HB fails to correct hallucinations effectively. Table 11 presents representative failure modes.

**Table 11:** Representative failure cases of HB. We categorize failures into false positives (unnecessary backtracking) and false negatives (missed hallucinations).

Failure type	Description	Analysis
False Positive	Original: “The beach has *golden sand* and blue water.” HB incorrectly triggers at “golden” despite sand being visible.	VAS drops due to abstract color description, but content is accurate. Threshold too sensitive for attribute descriptions.
False Negative	“A group of people at a party, **celebrating someone’s birthday with cake**.” Birthday cake not visible but VAS remains stable.	Hallucination based on scene inference rather than visual attention shift. Language priors dominate without detectable VAS change.
Repeated Failure	After 3 backtracks, model still generates “There is a **bicycle** near the car” when no bicycle exists.	Persistent hallucination from strong co-occurrence priors. Regeneration constraints insufficient for deeply embedded biases.

Our error analysis delineates three principal failure modes inherent to the current framework. First, *false Positives* (*12.3%* of detections) arise when legitimate declines in visual attention—often triggered by abstract concepts or transitional syntactic structures—are misclassified as hallucinations. This over-sensitivity precipitates unnecessary backtracking events, potentially introducing latency and interrupting the narrative flow. Second, *false Negatives* (*15.9%* of actual hallucinations) manifest when the model generates content derived from high-level scene priors rather than direct visual misinterpretation. In these instances, the VAS metric remains stable because the model’s internal representations treat the inferred content as consistent with the visual context, effectively bypassing the attention-based detection filter. Third, *repeated failures* occur in approximately *3.2%* of cases where the system exhausts the maximum backtracking budget ($\beta _{\max }=3$) without resolving the error. These recalcitrant cases are typically driven by potent linguistic co-occurrence priors that overpower visual constraints across multiple regeneration attempts. Collectively, these limitations chart a clear trajectory for future research, particularly highlighting the need for context-aware adaptive thresholding and multi-signal fusion strategies that augment VAS with complementary uncertainty indicators.

### Origin point distribution analysis

**Figure 8:**
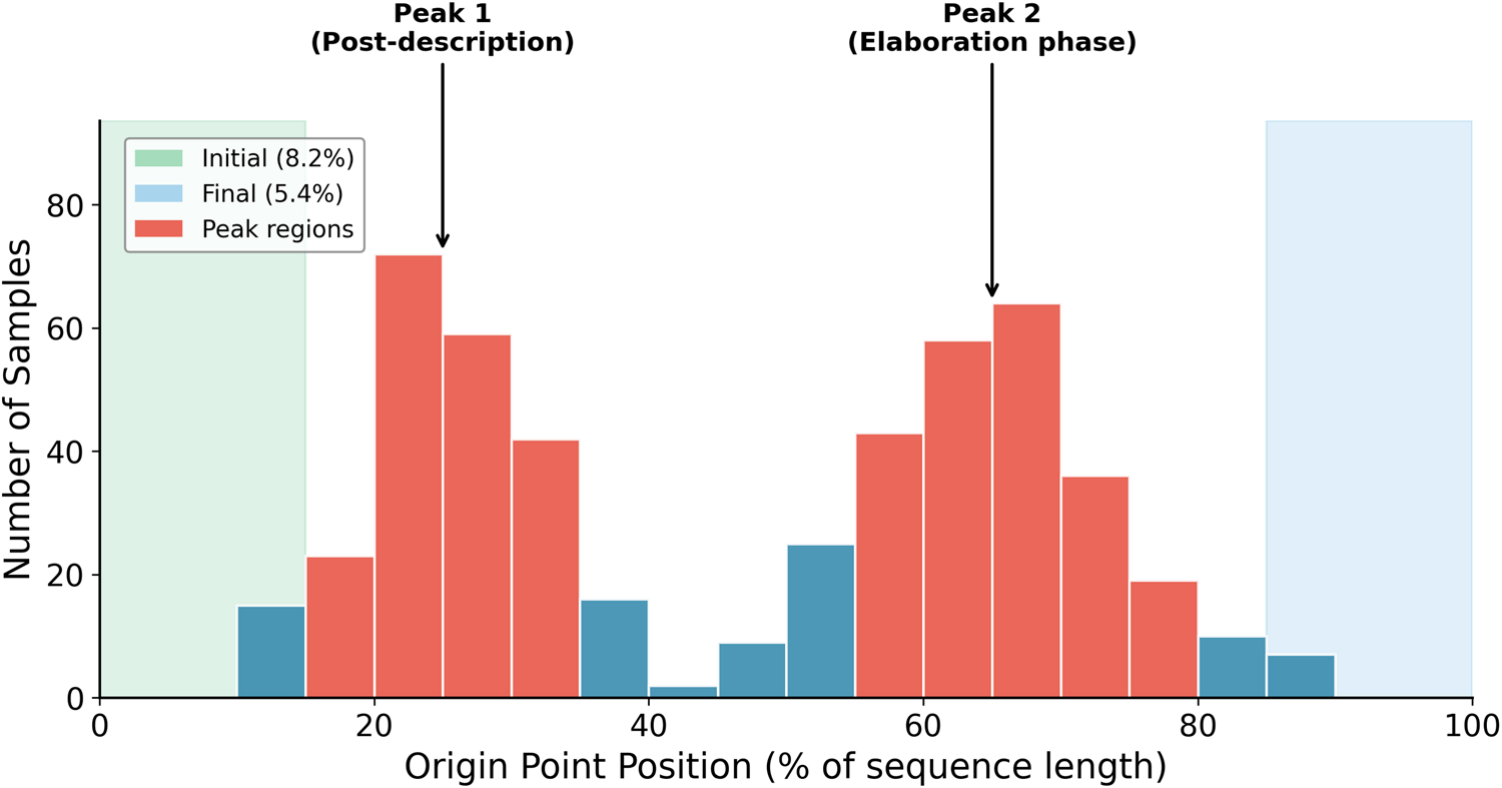
Distribution of hallucination origin points as percentage of total sequence length. The histogram reveals a bimodal pattern: Peak 1 (20–35%) corresponds to post-description transitions, Peak 2 (55–80%) corresponds to elaboration phases. The green and blue shaded regions indicate initial (8.2%) and final (5.4%) segments where hallucinations rarely originate.

To characterize the positional distribution of error introduction within the generation lifecycle, we analyzed the specific loci of hallucination onset across a corpus of 500 sequences compromised by confabulation. Figure 8 visualizes the temporal density of these detection points, revealing distinct structural tendencies regarding where deviations typically emerge within the narrative flow.

The empirical distribution underscores a non-uniform temporal susceptibility to hallucination, revealing four distinct phases within the generation lifecycle. First, the initial segment (0–15%) exhibits a low incidence rate (*8.2%*), suggesting that early generation is robustly anchored by high-salience visual features. Second, a primary peak emerges in the 20–30% interval, marking the transition from enumerating prominent entities to attributing secondary details; here, the system’s reliance begins to drift from visual evidence toward linguistic co-occurrence priors. Third, a subsequent spike in the 60–70% range corresponds to an “elaboration phase,” where the model attempts to extrapolate complex activities or latent relationships that exceed the bounds of explicit visual data. Finally, the terminal *10%* sees a return to stability (*5.4%*) as the generation converges toward syntactic closure and generalized summary statements, which demand less granular visual verification.

Table 12 analyzes the linguistic context of origin points by examining the part-of-speech tags of tokens immediately preceding the detected origin.

**Table 12:** Linguistic context analysis of hallucination origin points.

Preceding Context	Frequency	Example Pattern
After conjunction (and, with, while)	34.2%	“...walking the dog *and* [hallucination]”
After verb phrase	28.6%	“...is sitting *next to* [hallucination]”
After determiner (a, the, another)	18.9%	“There is also *a* [hallucination]”
After preposition	12.1%	“...on the table *with* [hallucination]”
Other	6.2%	Various patterns

**Figure 9:**
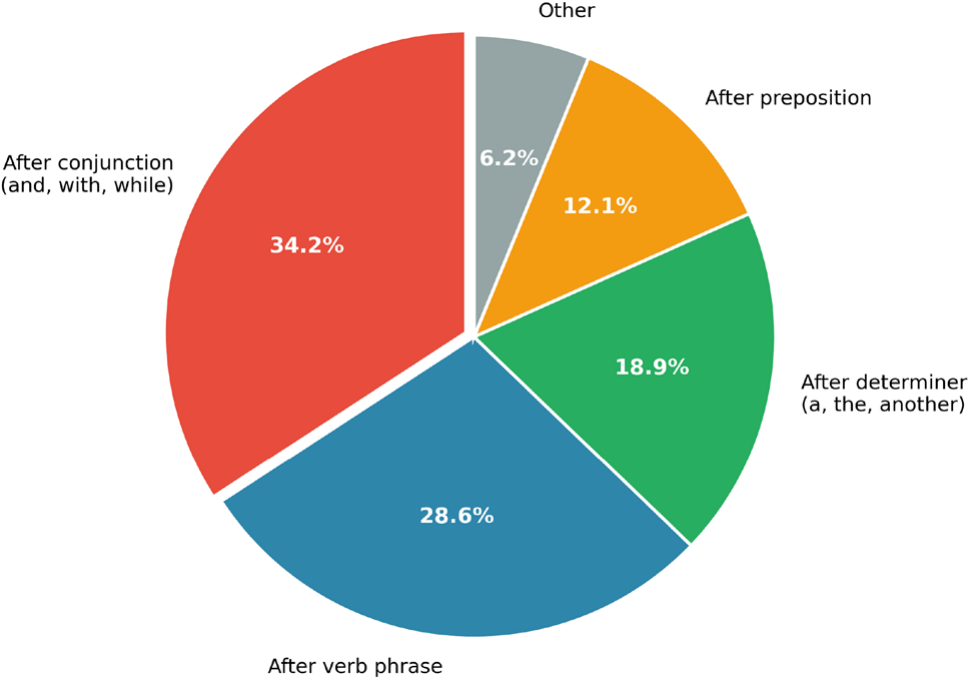
Linguistic context distribution of hallucination origin points. Conjunctions (34.2%) and verb phrases (28.6%) are the most common preceding contexts, indicating that additive and descriptive constructions are high-risk triggers for hallucination.

Syntactic analysis reveals a marked dependency between hallucination onset and specific grammatical markers, with conjunctions preceding *34.2%* of detected deviations—a distribution detailed in Fig. 9. This prevalence suggests that additive constructions (e.g., “and,” “with,” “also”) function as high-risk transitional states; in these contexts, the syntactic impetus to extend the narrative often outpaces the available visual evidence, leading the model to extrapolate beyond the image boundaries. This structural insight offers a strategic avenue for future optimization: specifically, the development of dynamic, context-aware detection thresholds that automatically heighten sensitivity during these syntactically vulnerable intervals.

## Discussion

Our results demonstrate that granular hallucination localization, facilitated by VAS, yields significantly more effective mitigation than coarse-grained global penalty mechanisms. The robustness of these improvements across diverse MLLM architectures and evaluation metrics indicates that the characteristic VAS decline constitutes a fundamental, model-agnostic precursor to hallucination onset, rather than an artifact of specific network topologies. The consistent advantage of HB over DeCo—a strong layer-contrastive baseline that operates along the vertical (inter-layer) axis—further substantiates that monitoring the cross-modal attention trajectory provides a more informative diagnostic signal than recovering suppressed knowledge from preceding layers, and that structural sequence correction outperforms distributional logit adjustments that preserve the corrupted context. Furthermore, our ablation study isolates the synergistic contributions of the proposed components: VAS provides a high-fidelity metric of cross-modal alignment that surpasses indirect proxies, while the backtracking mechanism ensures structural remediation—effectively removing the corrupted context to preclude the error propagation inherent to local, in-place correction strategies.

### Error taxonomy and detection boundaries

A candid assessment of the VAS-based detection paradigm requires a systematic examination of its failure modes, which delineate the structural boundaries of cross-modal attention monitoring as a single-signal diagnostic. Our error analysis identifies three distinct categories of failure, each illuminating a different facet of the gap between attention-based detection and the full complexity of the hallucination phenomenon.

The first category, *false negatives* (15.9% of actual hallucinations), represents instances where the model generates erroneous content without exhibiting a detectable VAS decline. These inference-driven hallucinations arise when the model synthesizes plausible content from high-level scene priors—for example, inferring the presence of a “birthday cake” at a “party” scene based on co-occurrence statistics. In such cases, the model’s internal representations treat the inferred content as consistent with the visual context, and the attention mechanism remains nominally engaged with the image even as the generated narrative departs from observable reality. This category arguably represents the most challenging type of hallucination for any attention-based detector, as the error originates not from a failure of visual grounding but from a failure of visual *interpretation*. Addressing these cases likely requires complementary signals beyond attention dynamics, such as semantic consistency verification or external knowledge grounding.

The second category, *false positives* (12.3% of detections), arises when legitimate declines in visual attention are misclassified as hallucination onsets. Our analysis reveals that these cases disproportionately occur during transitions from concrete visual description to abstract reasoning or functional attribution—for instance, when the model shifts from describing the physical appearance of an object to explaining its utility or contextual role. In such contexts, a reduction in visual attention reflects a natural cognitive transition rather than a pathological decoupling, yet the current static threshold cannot distinguish between the two. This observation suggests that the relationship between VAS magnitude and content fidelity is mediated by the semantic type of the generated content, a nuance that a uniform threshold inevitably overlooks.

The third category, *repeated failures* (3.2% of cases), occurs when the backtracking mechanism exhausts its budget ($\beta _{\max }=3$) without resolving the hallucination. These recalcitrant cases are typically driven by deeply embedded co-occurrence priors that consistently override visual constraints across multiple regeneration attempts, indicating a class of errors where inference-time intervention alone may be insufficient without complementary training-time alignment.

Collectively, these failure modes underscore that VAS captures one—albeit highly informative—dimension of the multifaceted hallucination phenomenon. The 84.1% before-first detection accuracy confirms that cross-modal attention decline is the dominant precursor to hallucination, but the residual 15.9% false negative rate demonstrates that a comprehensive mitigation framework would benefit from multi-signal fusion strategies that augment attention-based monitoring with complementary indicators such as token-level uncertainty, semantic coherence scores, or layer-wise consistency metrics.

### On the static threshold and task-dependent attention dynamics

The current framework employs a global static threshold (*τ =0.15*) for hallucination detection, calibrated to balance sensitivity and specificity across the evaluated architectures. While our sensitivity analysis demonstrates robust performance within the interval $\tau \in [0.10, 0.20]$ (Fig. 4), the assumption of a fixed threshold warrants critical examination, particularly regarding its behavior under varying task demands.

Visual attention allocation is inherently task-dependent: the distribution of attention across visual and textual tokens shifts substantially based on the specific instructional demand. A descriptive prompt (“describe this image in detail”) naturally elicits sustained visual consultation throughout the generation process, whereas a narrative prompt (“tell me a story about this image”) may legitimately shift toward language-driven elaboration after an initial anchoring phase. Similarly, enumerative prompts (“list the objects here”) and reasoning prompts (“what might happen next?”) are likely to produce distinct baseline VAS profiles. Consequently, a threshold calibrated under descriptive prompting conditions may exhibit degraded specificity under prompts that naturally induce lower baseline VAS values—potentially increasing false positive rates—or degraded sensitivity under prompts that maintain artificially high VAS through repeated visual reference.

Our linguistic context analysis (Table 12) provides empirical evidence that attention dynamics are indeed modulated by syntactic structure: conjunctions preceding 34.2% of hallucination onsets suggests that additive constructions create syntactically vulnerable intervals where detection sensitivity should be heightened. This observation motivates the development of context-aware dynamic thresholding, where *τ* is modulated in real-time based on the syntactic and semantic properties of the generation context. Concretely, one could envision a threshold function $\tau (t) = \tau _0 + f(\text {POS}_{t-1}, H_{\text {syn}}(t))$, where $\tau _0$ is a base threshold and *f* adjusts sensitivity based on the preceding part-of-speech tag and the local syntactic entropy $H_{\text {syn}}$. We identify this as a high-priority direction for future work.

We acknowledge that our primary evaluation of the CHAIR metric relies on a single prompt template (“Please describe this image in detail”), which constrains the empirical assessment of prompt sensitivity. While the POPE and AMBER benchmarks employ distinct query formats that partially broaden the evaluation scope, a systematic multi-prompt evaluation remains necessary to fully characterize the robustness of the detection mechanism across diverse instructional contexts.

### Max-pooling aggregation: rationale and boundaries

The selection of max-pooling as the aggregation strategy for synthesizing attention weights across heads is grounded in the well-documented phenomenon of functional specialization within multi-head attention mechanisms. Interpretability studies on transformer architectures have demonstrated that individual attention heads develop distinct functional roles^32,33^ , with specific heads specializing in syntactic dependencies, positional tracking, or—in the multimodal context—cross-modal grounding. Under this framework, the max operator serves as a sufficient condition detector: if any single head maintains a strong visual connection, the model retains access to visual information through that channel, and the VAS should reflect this capacity.

Our empirical results support this rationale: max-aggregation yields a 2.2-point F1 improvement and a 3.6-point CHAIR$_S$ reduction over mean aggregation (Table 6), confirming that peak attention magnitude is a more reliable predictor of factual integrity than the diluted average. However, we acknowledge an important theoretical caveat: a high attention weight from a single “visual specialist” head constitutes a *necessary but not sufficient* condition for faithful generation. The attention weight quantifies the degree to which information from visual tokens is *read* into the current representation, but it does not guarantee that this information is correctly *integrated* into the downstream semantic computation across subsequent transformer layers. It is conceivable that a head attends strongly to the correct visual region yet the resulting representation is overridden by competing signals in later processing stages.

Establishing a definitive causal link between the activity of specific attention heads and the factual content of the generated output would require targeted interpretability analyses—such as attention head ablation studies or probing classifiers applied to individual head outputs—that lie beyond the scope of this work. We note, however, that the strong empirical correlation between max-VAS decline and hallucination onset (84.1% before-first accuracy) provides substantial practical validation, even in the absence of a fully articulated causal mechanism. Future work could explore more sophisticated aggregation strategies, such as head importance weighting^32^ or layer-wise gating mechanisms, that move beyond the binary logic of max-pooling to capture more nuanced patterns of visual information flow.

### Scope of the training-free paradigm

The characterization of HB as “training-free” merits precise delineation. We define this property as the absence of any gradient-based parameter updates or task-specific fine-tuning applied to the underlying MLLM; the model weights remain entirely frozen throughout the inference process. Under this definition, HB operates strictly as a decoding-time intervention, requiring no additional training data, reward models, or auxiliary networks.

However, the VAS-guided sampling component (described in the “Methods” section) employs a first-order approximation that projects candidate embeddings into a “visual relevance subspace” derived from a calibration set of 500 MSCOCO images. This calibration step, while lightweight and performed offline, introduces a form of data dependence that warrants transparent acknowledgment. The calibration procedure extracts principal components from the visual token representations of these images to construct a subspace that captures the dominant directions of visual relevance; candidate tokens are then scored based on their alignment with this subspace.

Several considerations mitigate the concern that this calibration step undermines the generalizability of the framework. First, the subspace is derived from the model’s own visual representations rather than from external annotations, and its construction involves only linear algebra operations (PCA) with no gradient computation. Second, the subspace captures generic properties of visual token geometry that reflect the encoder’s representational structure rather than dataset-specific content; preliminary analysis indicates that the principal components stabilize with as few as 300 images, suggesting low sensitivity to the specific calibration samples. Third, the approximation operates under a bounded error regime, matching the true *Δ*VAS within a margin of 0.05 in 89% of cases.

Nevertheless, we recognize that deployment in visual domains substantially different from natural images (e.g., medical imaging, satellite imagery, or technical diagrams) may necessitate domain-specific recalibration to ensure that the visual relevance subspace accurately reflects the geometric properties of the target domain. We consider the development of domain-adaptive or calibration-free alternatives—for instance, online subspace estimation during inference—as a valuable direction for future research.

### Limitations and future directions

Beyond the specific methodological boundaries discussed above, several broader limitations merit acknowledgment. First, the framework is specifically designed for long-form generative tasks, such as dense captioning, where the temporal dynamics of attention drift provide sufficient signal for detection ; its utility may be attenuated in short-answer VQA contexts where generation lengths are too brief for meaningful VAS trajectory analysis. Second, the active monitoring and backtracking mechanisms introduce computational latency compared to standard decoding strategies, though as discussed in our efficiency analysis, the incremental overhead relative to the underlying beam search paradigm remains modest. Third, the current evaluation focuses on English-language generation with natural image inputs; the generalizability to multilingual settings or non-photographic visual domains remains to be established.

This research charts several promising avenues for future exploration. First, we envision the synergistic integration of VAS-based inference-time monitoring with training-centric alignment methods^5,6^, potentially creating a hybrid framework that combines intrinsic model robustness with extrinsic safety guardrails. Second, there is significant potential in extending this paradigm to spatiotemporal domains, such as video understanding and multi-image reasoning, where temporal attention consistency offers a richer signal for error detection. Third, the development of context-aware dynamic thresholding—where detection sensitivity is modulated based on syntactic context, semantic entropy, and task-specific priors—represents a natural evolution of the current static threshold approach. Fourth, multi-signal fusion strategies that combine VAS with complementary indicators (e.g., layer-wise consistency à la DeCo^22^ , token-level uncertainty, or semantic coherence scores) could address the inference-driven hallucinations that currently evade attention-based detection. Finally, given its training-free architecture, HB offers immediate interoperability with emerging foundation models, serving as a modular verification layer particularly suited for safety-critical applications requiring verifiable visual grounding^26^.

## Conclusion

In this paper, we introduced HB, a novel, training-free decoding framework engineered to mitigate object and attribute fabrications in MLLMs. The efficacy of our approach is predicated on a fundamental empirical insight: that hallucination is not a global failure, but typically originates at discrete token boundaries where the model’s attention mechanisms decouple from visual stimuli in favor of strong autoregressive linguistic priors. By rigorously isolating these specific loci of divergence, HB enables precise intervention—correcting errors at their genesis while preserving the integrity and fluency of the surrounding veridical content.

The operational linchpin of our framework is VAS, a token-level metric engineered to quantify the fidelity of cross-modal alignment during the decoding sequence. Diverging from prevalent methodologies that depend on indirect proxies—such as aggregated attention on global summary tokens—VAS delivers an explicit, real-time gauge of visual grounding. This granular resolution enables the continuous mapping of attention dynamics throughout the generative process. Crucially, our empirical validation establishes that a characteristic decay in VAS serves as a robust precursor to hallucination onset, offering a reliable signal for precisely localizing the point where the model’s focus drifts from visual reality.

Leveraging the diagnostic precision of VAS, we formulated a backtracking-and-regeneration mechanism that represents a methodological departure from standard penalty-based decoding. Unlike approaches that rely on local logit suppression—effectively attempting to modulate probability distributions while retaining the corrupted context—HB executes a structural intervention. By truncating the generation trajectory at the precise locus of divergence and re-initiating decoding with reinforced visual constraints, the framework addresses the contextual root of the hallucination. This strategy enables more effective remediation, mitigating the downstream error propagation often observed in methods that merely adjust token probabilities without removing the source.

Extensive experiments across a diverse suite of hallucination benchmarks (POPE, CHAIR, and AMBER) and four distinct MLLM architectures (LLaVA-1.5, InstructBLIP, MiniGPT-4, and Shikra) substantiates that HB consistently surpasses state-of-the-art inference-time baselines, including DeCo, OPERA, and VCD. Complementary ablation studies validate the synergistic efficacy of both the VAS-driven detection logic and the regenerative backtracking mechanism, while granular localization analysis confirms that HB identifies the genesis of hallucinations with significantly higher precision than alternative detection methodologies. Crucially, HB realizes these performance gains within a strict training-free paradigm; by necessitating no additional parameter optimization, external datasets, or auxiliary networks, the framework ensures immediate, plug-and-play applicability to current and future pre-trained MLLM foundations.

We acknowledge that the current framework operates within well-defined boundaries. The VAS-based detection mechanism, while effective for the dominant class of attention-driven hallucinations, does not capture inference-driven fabrications where the model generates erroneous content without a detectable shift in visual attention. The static detection threshold, the reliance on a single aggregation strategy, and the scope of the current evaluation across prompt types represent areas where further refinement is warranted, as discussed in detail in the preceding section.

This work advances a distinct paradigm for mitigating MLLM hallucinations, prioritizing the rigorous localization and targeted correction of error origins over generalized suppression strategies. We posit that this shift toward identifying the precise genesis of failure—rather than relying on coarse, global interventions—constitutes a promising trajectory for improving the reliability of multimodal systems, particularly within the exacting demands of safety-critical deployment.

## Data Availability

The MSCOCO dataset is available at https://cocodataset.org. The POPE benchmark is available at https://github.com/AoiDragon/POPE. The AMBER benchmark is available at https://github.com/junyangwang0410/AMBER. Code availability: The code is available from the corresponding author upon reasonable request.

## Code availability

The code is available from the corresponding author upon reasonable request.
